# Crystal and Magnetic Structures of the Ternary Ho_2_Ni_0.8_Si_1.2_ and Ho_2_Ni_0.8_Ge_1.2_ Compounds: An Example of Intermetallics Crystallizing with the Zr_2_Ni_1–*x*_P Prototype

**DOI:** 10.1021/acs.inorgchem.1c02211

**Published:** 2021-10-15

**Authors:** Alessia Provino, Clemens Ritter, Volodymyr Smetana, Anja-Verena Mudring, Marcella Pani, Vitalij K. Pecharsky, Pietro Manfrinetti

**Affiliations:** ‡ Department of Chemistry, University of Genova, 16146 Genova, Italy; § The Ames Laboratory, U.S. Department of Energy, Iowa State University, Ames, Iowa 50011-3020, United States; ⊥ Institut Laue-Langevin (ILL), 38042 Grenoble, France; ∥ Department of Materials and Environmental Chemistry, Stockholm University, 10691 Stockholm, Sweden; ¶ Institute SPIN-CNR, 16152 Genova, Italy; # Department of Materials Sciences and Engineering, Iowa State University, Ames, Iowa 50011-2300, United States

## Abstract

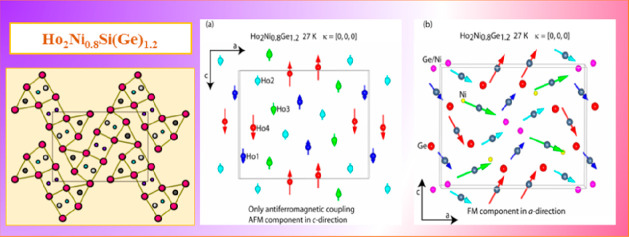

We report two new rare-earth (R) ternary intermetallic compounds—Ho_2_Ni_0.8_T_1.2_ with T = Si and Ge—that correspond to the R_5_Ni_2_T_3_ phase earlier reported to form in Dy–Ni–T and Ho–Ni–T ternary systems. The compounds crystallize in a filled version of the orthorhombic Zr_2_Ni_1–*x*_P-type structure with *x* = 0.52; their stoichiometry, determined from both single-crystal and powder X-ray diffraction data, is centered on Ho_2_Ni_0.8_T_1.2_ with a narrow solid solubility range for the silicide, while the germanide appears to be a line phase. In addition to R = Dy and Ho, R_2_Ni_0.8_T_1.2_ compounds also form for R = Y and Tb, representing the first examples of rare-earth-based compounds adopting the Zr_2_Ni_1–*x*_P structural prototype. Bulk magnetization data reveal the main transitions of the ferrimagnetic or ferromagnetic type at *T*_C_ = 38 K for Ho_2_Ni_0.8_Si_1.2_ and *T*_C_ = 37 K for Ho_2_Ni_0.8_Ge_1.2_, which are followed by subsequent magnetic reordering at lower temperatures. Neutron diffraction shows complex magnetic structures below *T*_C_ with both ferromagnetic and antiferromagnetic components and magnetic propagation vector κ_1_ = [0, 0, 0]. Below *T*_N_ ≅ 24 K (22 K) for the silicide (germanide), an additional antiferromagnetic coupling following an incommensurate magnetic propagation vector κ_2_ = [κ_*x*_, 0, 0] appears to coexist with the first magnetic structure.

## Abstract 2

The crystal structure of Ho_2_Ni_0.8_Si_1.2_ (and Ho_2_Ni_0.8_Ge_1.2_) [Zr_2_Ni_0.48_P-type (*oP*32-*Pnma*)] viewed along the *b* axis is given. The Ho, Ni1, Ni2/Si, Si1 and Si2 atoms are represented as red, turquoise, blue, light-gray, and dark-gray spheres, respectively. The distinctive structural fragment is built up of five differently oriented trigonal prisms. The magnetic structure of Ho_2_Ni_0.8_Ge_1.2_ with the antiferromagnetic component in the *c* direction and the ferromagnetic component along the *a* axis.

## 1 Introduction

In addition to supporting continuous developments in industry, especially in high-technology areas, advanced materials help to save energy and reduce deleterious effects on the environment, thus improving standards of living. Among many classes of different materials, intermetallic compounds represent a vast and excellent resource, many of them with a strong potential to be deployed in various technological applications. Of note, R-based compounds, where R = rare earth, remain among the most interesting and investigated because of the emergence of unique, sometimes exotic, properties and functionalities brought about by the R atoms and their peculiar electronic structures, which also makes them crucial materials for many technologies.^1^

During our earlier exploration of new ternary phases in the Dy–Ni–Si^2^ and Ho–Ni–Ge^3^ systems, we identified intermetallics forming with the approximate compositions of R_5_Ni_2_T_3_, where T = Si or Ge. At the time, the crystal structures, exact compositions, and properties of these two new compounds remained unknown. More recently, the same phase has been reported to form also in the Ho–Ni–Si ternary system, however, still without an investigation and determination of its crystal structure.^4^

In this work, we establish the crystal structures of the “Ho_5_Ni_2_T_3_” compounds for T = Si and Ge and found them to crystallize in an orthorhombic unit cell whose prototype is Zr_2_Ni_1–*x*_P [space group *Pnma* (No. 62); Pearson symbol *oP*32–y)].^5^ Therefore, the true stoichiometry is not R_5_Ni_2_T_3_ but very close to it, namely, R_2_Ni_1–*x*_T_1+*x*_, with 0.094(1) ≤ *x* ≤ 0.250(1) for Ho_2_Ni_1–*x*_Si_1+*x*_ and 0.190(1) ≤ *x* ≤ 0.201(1) for Ho_2_Ni_1–*x*_Ge_1+*x*_. Noting that no other rare-earth–transition-metal silicides or germanides have ever been reported to crystallize with the Zr_2_Ni_1–*x*_P prototype,^6−9^ we prepared homologues with other heavy lanthanides, finding that the phase with R = Gd does not form, while it forms when R = Y, Tb, Dy, and Ho. In addition to the formation and crystal structure of these new R_2_Ni_1–*x*_T_1+*x*_ ternary intermetallic compounds, we report physical properties and magnetic structures of the two Ho compounds at the Ho_2_Ni_0.8_T_1.2_ composition for T = Si and Ge.

## 2 Experimental Methods

### 2.1 Synthesis, Phase, and Crystallographic Analyses

Polycrystalline samples (for R = Gd, Tb, Dy, Ho, and Y and T = Si and Ge) were prepared by arc melting from the constituent elements, weighed in stoichiometric amounts, under a pure TiZr-gettered Ar atmosphere. The purity of the rare-earth metals was 99.9+ wt % with respect to all other elements in the periodic table (the metals were prepared by the Materials Preparation Center of the Ames Laboratory).^10^ The purities of non-rare-earth elements purchased commercially were 99.99 wt % for Ni and 99.999 wt % for Si and Ge. The total mass of the samples was 3–4 g for crystallographic investigation and physical property measurements and 7–8 g for neutron diffraction investigation. The buttons were melted three times, turning them upside down after each melting to ensure homogenization. Weight losses were less than 0.7 wt % (less than 0.3 wt % for the samples for neutron diffraction). Particular care was taken during the melting and cooling processes [by preventively avoiding strong cooling of copper hearth by adopting a low cooling-water flow, performing a progressive and slow heating and melting process under a minimal heating direct-current (dc) electric current of the arc, and slowly lowering the temperature of the sample by progressively reducing the power/current at the end of melting, while avoiding quick break of power] because the alloys are sensitive to thermal shock, with a strong tendency to shatter into pieces. Initially, samples for the crystal structure determination, for phase analysis, and for the checking of limits of solid solubility were prepared for R = Ho with three nominal compositions: Ho_50_Ni_18.3_T_31.7_, Ho_50_Ni_20_T_30_, and Ho_50_Ni_25_T_25_ for both T = Si and Ge. Later, samples with the nominal compositions Ho_50_Ni_20_T_30_ (T = Si and Ge) were prepared for neutron diffraction investigation. The as-prepared ingots were wrapped in a Ta foil and sealed under vacuum in a quartz tube. They were annealed at 1000 °C for 4–7 days followed by air cooling to room temperature after the ampoules were taken out of the furnace.

The microstructure and homogeneity of the alloys were checked by light optical and scanning electron (SEM) microscopies, with the latter performed on an instrument equipped with an energy-dispersive X-ray (EDX) microprobe for semiquantitative elemental analysis [a Leica Cambridge 360 microscope, equipped with an Oxford X-Max 20 analyzer; work parameters: EHT 20.0 kV and probe current 220 pA (Oxford *Aztec* software)]. EDX analyses were performed on at least four sample points (or areas) to identify the phase composition, with a counting time of 60 s. Extra phases present as impurities (i.e., Ho_3_NiT_2_ and HoNiT) were used as internal standards; the precision of the measurements was estimated to be within 1 atom %. SEM images of the samples were taken using both backscattered and secondary-electron modes.

A PANanalytical X’Pert diffractometer (Cu Kα_1_ radiation) and a Guinier camera [Cu Kα_1_ radiation with Si as an internal standard; *a* = 5.4308(1) Å] were used to collect the powder X-ray diffraction (XRD) data; the Guinier patterns were indexed with *LAZY PULVERIX*,^11^ and accurate lattice parameters were obtained by least-squares refinement. The Rietveld refinements were carried out by using the *FullProf* program.^12^ The single-crystal XRD was performed at room temperature on either a Bruker Apex CCD diffractometer or a Bruker D8 Venture diffractometer (both with Mo Kα radiation), utilizing the *APEX2* and *APEX3* software suites (for the former and latter diffractometers, respectively) for data collection between 2 and 60° of 2θ, integration, polarization, and empirical absorption correction.^13,14^ The *SHELXTL* suite and *XPREP* algorithms were used to check for extinction conditions and *E* statistics in the intensity data sets necessary for assignment of the proper *Pnma* space group. Direct methods were used for structure solution (*SHELXS-97*).^15^*APEX3* software was then used to carry out full structure refinement (determining atomic positions, mixed site occupancies, and anisotropic displacement parameters).

### 2.2 Thermal Analysis

Differential thermal analysis (DTA) was performed by using a Netzsch 404 thermal analyzer on bulk samples of 0.7–0.9 g, either as-cast or annealed, sealed in an outgassed Mo crucible under an Ar atmosphere. Data were recorded upon heating at 20 K/min and upon cooling at 10 K/min (temperature measurement accuracy ±5 K). The results obtained from DTA were, however, inconclusive for both Ho_2_Ni_0.8_Si_1.2_ and Ho_2_Ni_0.8_Ge_1.2_, making it impossible to establish how both compounds form or melt/decompose; it is likely that their formation/melting or decomposition temperatures are higher than the equipment limit of 1650 °C.

### 2.3 Physical Property Measurements

The magnetization measurements were carried out using a Magnetic Property Measurement System (SQUID, Quantum Design). The measurements were performed on the samples prepared in the middle of the solid solubility range of the two Ho_2_Ni_1–*x*_Si_1+x_ and Ho_2_Ni_1–*x*_Ge_1+*x*_ phases; these turned out to be single phases with compositions close to Ho_2_Ni_0.8_Si_1.2_ and Ho_2_Ni_0.8_Ge_1.2_ (averaged data from the EDX microprobe and Rietveld refinement; Table 3). The dc magnetization as a function of the temperature was measured in both zero-field-cooled (ZFC) and field-cooled (FC) modes between 2 and 300 K and under several applied magnetic fields. The isothermal magnetization was measured at various temperatures in applied fields up to 70 kOe for both compounds. Heat capacity data were collected between 2 and 100 K in both zero and applied magnetic fields using a home-built automated semiadiabatic calorimeter.^16^

### 2.4 Neutron Diffraction Measurements

The neutron diffraction investigations were performed at the ILL, Grenoble, France, using the high-resolution powder diffractometer D2B (λ = 1.594 Å) and the high-intensity powder diffractometer D1B (λ = 2.52 Å). The temperature dependencies of the powder neutron diffraction patterns (thermodiffractograms) were measured on D1B between 1.5 and 40 K for the Si compound and between 1.5 and 46 K for the Ge compound by taking data at Δ*T* = 1.2 K intervals. High-resolution data were taken on D2B at 300 K for both compounds using the additional 10′ collimation of the primary beam. Data analysis was performed using the Rietveld refinement program *FullProf*;^12^ magnetic symmetry analysis was performed using the program *BASIREPS*.^17,18^

## 3 Results and Discussion

### 3.1 Crystal Structure of the R_2_Ni_0.8_T_1.2_ Compounds

Crystallites suitable for single-crystal XRD examination were selected from a sample with nominal composition Ho_50_Ni_20_Si_30_. We found that the structural prototype of this and all other new compounds is the orthorhombic Zr_2_Ni_1–*x*_P [*Pnma* (No. 62); *oP*32–y].^5^ The prototypical phosphide is Ni-deficient, forming at *x* = 0.52, which yields a composition Zr_2_Ni_0.48_P. The Zr_2_Ni_0.48_P structure features eight independent 4*c* Wyckoff sites, four of which are occupied by the largest Zr atoms, two by the Ni atoms, and two by the smallest P atoms. Both of the Ni sites are partially occupied, at 19% and 76%. Unlike in the Zr_2_Ni_0.48_P prototype, all of the 4*c* Wyckoff sites are fully occupied in Ho_50_Ni_20_T_30_ when T = Si or Ge, with one of the four non-rare-earth atom sites clearly showing mixed occupancy by Ni and Si and another possibly a minor mixing of Ni of the site predominantly occupied by Si. The final refined composition for T = Si is Ho_50_Ni_19.2(1)_Si_30.8(1)_, which corresponds to the Ho_2_Ni_0.769(5)_Si_1.231(5)_ formula unit, hence suggesting that the correct stoichiometry of these new silicides and germanides, previously reported as R_5_Ni_2_T_3_, should be R_2_Ni_1–*x*_T_1+*x*_. The details of single-crystal analysis for the Ho_2_Ni_0.769(5)_Si_1.231(5)_ compound are shown in Table S1, the refined atomic coordinates, site occupancies, and isotropic displacement parameters are listed in Table 1, and the anisotropic displacement parameters are found in Table S2. As in the prototype, the unit cell accommodates eight 4*c* Wyckoff positions: four of them are fully occupied by the larger Ho atoms and four by the smaller Ni and Si atoms, including one site populated by a statistical mixture of Ni and Si in nearly equal ratio (52% Ni + 48% Si) and another that is mostly populated by Si atoms (98% Si + 2% Ni). Consequently, and for the sake of simplicity, in the following discussion, full Si occupancy is assumed for the Si1 (Table 1) site. The interatomic distances and coordination numbers for all atoms are reported in Table 2.

**Table 1:** Standardized Fractional Atomic Coordinates and Isotropic Displacement Parameters for Ho_2_Ni_0.769(5)_Si_1.231(5)_ [*oP*32; *Pnma* (No. 62)]^a^

atom		atomic coordinates			occupation	
Zr_2_Ni_0.48_P	Ho_2_Ni_0.769(5)_Si_1.231(5)_	*x*	*z*	*U*_iso_ [Å^2^]	Zr_2_Ni_0.48_P	Ho_2_Ni_0.769(5)_Si_1.231(5)_
Zr1	Ho1	0.03409(2)	0.78768(4)	0.0068(1)	1	1
Zr2	Ho2	0.14664(3)	0.09384(4)	0.0068(1)	1	1
Zr3	Ho3	0.27026(3)	0.35569(4)	0.0069(1)	1	1
Zr4	Ho4	0.39340(3)	0.03006(4)	0.0058(1)	1	1
Ni1	Ni1	0.34860(8)	0.7825(1)	0.0074(2)	0.19(6)	1
Ni2	Ni2/Si	0.4575(1)	0.4723(1)	0.0072(5)	0.76(2)	0.516(9)/0.484(9)
P1	Si1/Ni	0.0714(2)	0.3435(2)	0.0075(7)	1	0.978(9)/0.022(9)
P2	Si2	0.2148(2)	0.6561(2)	0.0072(5)	1	1

a *U*_eq_ is defined as one-third of the trace of the orthogonalized *U*_*ij*_ tensor. The crystallographic data of the prototype Zr_2_Ni_0.48_P [*oP*32–y; *Pnma* (No. 62)] are also reported for comparison. All atoms are in the 4*c* Wyckoff position (*x*, ^1^/_4_, *z*).

**Table 2:** Interatomic Distances in Ho_2_Ni_0.769(5)_Si_1.231(5)_ [*oP*32; *Pnma* (No. 62)]^a^

central atom	ligand	*d*_obs_ [Å]	central atom	ligand	*d*_obs_ [Å]
Ho1 (CN = 17)	1 Ni1	2.868(1)	Ho2 (CN = 17)	2 Ni2/Si	2.899(1)
	1 Ni2/Si	2.887(1)		1 Ni2/Si	2.908(2)
	2 Ni2/Si	2.8938(8)		2 Ni1	2.9236(8)
	2 Si1/Ni	2.960(2)		1 Si1/Ni	2.977(2)
	1 Si2	3.058(3)		2 Si2	2.987(2)
	2 Ho2	3.6256(5)		1 Ho3	3.4292(6)
	2 Ho3	3.6386(4)		2 Ho3	3.5570(5)
	2 Ho4	3.6696(5)		2 Ho1	3.6256(5)
	1 Ho2	3.7755(6)		1 Ho4	3.7396(6)
	1 Ho4	4.0884(5)		1 Ho1	3.7755(6)
	2 Ho1	4.0965(3)		2 Ho2	4.0965(3)
Ho3 (CN = 17)	2 Ni1	2.8247(9)	Ho4 (CN = 17)	1 Ni1	2.816(1)
	1 Si1/Ni	2.963(3)		2 Si1/Ni	2.953(2)
	2 Si2	3.018(2)		2 Si2	2.954(2)
	1 Ni2/Si	3.070(2)		1 Si1/Ni	2.995(3)
	1 Si2	3.421(2)		2 Ho1	3.6696(5)
	1 Ho2	3.4292(6)		2 Ho3	3.7204(5)
	2 Ho2	3.5570(5)		1 Ho2	3.7396(6)
	2 Ho1	3.6386(4)		2 Ho4	3.8347(5)
	2 Ho4	3.7204(5)		1 Ho3	4.0385(6)
	1 Ho4	4.0385(6)		1 Ho1	4.0884(5)
	2 Ho3	4.0965(3)		2 Ho4	4.0965(3)
Ni1 (CN = 9)	1 Si2	2.432(3)	Ni2/Si (CN = 9)	2 Ni2/Si	2.484(1)
	2 Si1/Ni	2.463(2)		1 Ho1	2.887(1)
	1 Ho4	2.816(1)		2 Ho1	2.8938(8)
	2 Ho3	2.8247(9)		2 Ho2	2.899(1)
	1 Ho1	2.868(1)		1 Ho2	2.908(2)
	2 Ho2	2.9236(8)		1 Ho3	3.070(2)
Si1/Ni (CN = 9)	2 Ni1	2.463(2)	Si2 (CN = 9)	1 Ni1	2.432(3)
	2 Ho4	2.953(2)		2 Ho4	2.954(2)
	2 Ho1	2.960(2)		2 Ho2	2.987(2)
	1 Ho3	2.963(3)		2 Ho3	3.018(2)
	1 Ho2	2.978(2)		1 Ho1	3.058(3)
	1 Ho4	2.995(3)		1 Ho3	3.421(3)

a Values are within *d*_obs_/∑*r* ≤ 1.16, with ∑*r* the sum of the involved metallic radii for coordination 12.

SEM images, representative of the microstructure of the samples with starting nominal compositions Ho_50_Ni_18.3_Si_31.7_, Ho_50_Ni_20_Si_30_, and Ho_50_Ni_25_Si_25_ are shown in parts a–c of Figure S1, respectively. The main phase in the three samples has compositions of Ho_50(1)_Ni_18(1)_Si_31(1)_, Ho_50(1)_Ni_20(1)_Si_30(1)_ (matching the stoichiometry refined from the single-crystal XRD data well), and Ho_50(1)_Ni_22(1)_Si_28(1)_, respectively. The results of microprobe analysis, therefore, indicate that the Ho_2_Ni_1–*x*_Si_1+*x*_ compound is stable over a certain range of *x*.

**Figure 1:**
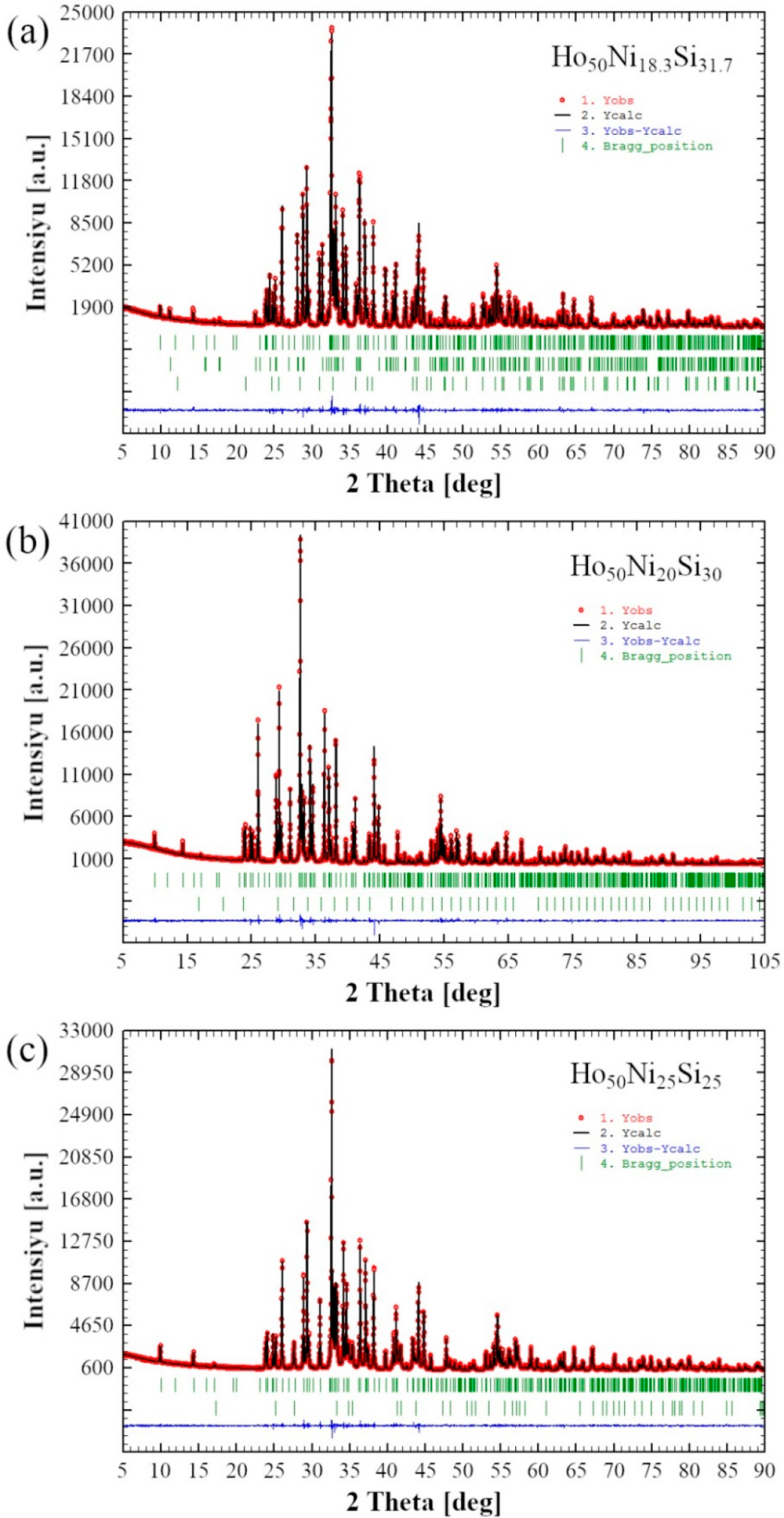
Observed (red circles) and calculated after the Rietveld refinements (black lines) intensities of the powder XRD patterns for nominal samples: (a) Ho_50_Ni_18.3_Si_31.7_, which in addition to the main Ho_2_Ni_0.750(1)_Si_1.250(1)_ phase (upper row of vertical bars indicating the calculated Bragg peak positions) contains Ho_3_NiSi_2_ (middle row) and Ho_5_Ni_0.20(4)_Si_2.80(4)_ (lower row) impurities; (b) Ho_50_Ni_20_Si_30_ containing Ho_2_Ni_0.846(1)_Si_1.140(1)_ (main phase, upper row) and Ho_2_O_3_ (impurity, lower row); (c) Ho_50_Ni_25_Si_25_ containing Ho_2_Ni_0.922(3)_Si_1.078(3)_ (main phase, upper row) and HoNi_0.855(2)_Si_0.145(2)_ (impurity, lower row). The blue lines at the bottom of each plot are the differences between the observed and calculated intensities.

**Figure 2:**
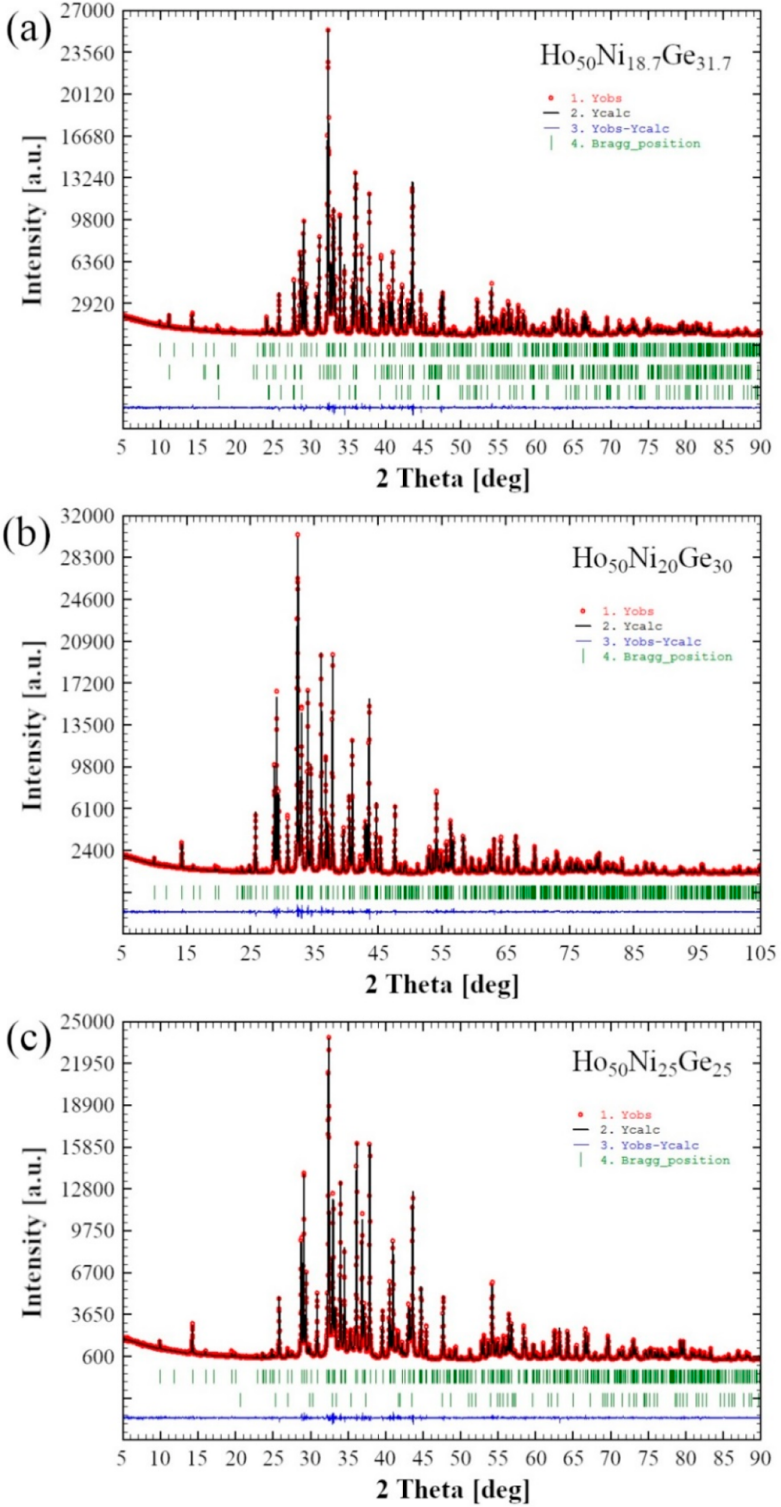
Observed (red circles) and calculated after the Rietveld refinements (black lines) intensities of the powder XRD patterns for nominal samples: (a) Ho_50_Ni_18.3_Ge_31.7_, which in addition to the main Ho_2_Ni_0.799(1)_Ge_1.201(1)_ phase (upper row of vertical bars indicating the calculated Bragg peak positions) contains Ho_3_NiGe_2_ (middle row) and HoNiGe (lower row); (b) Ho_50_Ni_20_Ge_30_ (a single-phase sample); (c) Ho_50_Ni_25_Ge_25_ containing Ho_2_Ni_0.810(1)_Ge_1.190(1)_ (upper row) and HoNi_0.925_Ge_0.075_ (lower row). The blue lines at the bottom of each plot are the differences between the observed and calculated intensities.

The Rietveld refinements of the powder XRD patterns of all prepared samples with R = Ho are shown in Figures 1 and 2, fully corroborating the results obtained from SEM–EDX for the silicides.

Crystallographic data obtained from the Rietveld refinements, both for the main phase and for the detected impurity phase(s), are collected in Tables S3–S8. The nominal compositions, the resulting compositions of the main Ho_2_Ni_1–*x*_T_1+*x*_ phase from EDX analyses, and the Rietveld-refined stoichiometries along with the lattice parameters, observed unit cell volumes (*V*_obs_), and volume contractions (ΔV), are collected in Table 3. The volume contraction during the formation of a compound is defined as Δ*V* % = [(*V*_obs_ – *V*_calc_)/*V*_calc_] × 100, where *V*_calc_ is the cell volume calculated from the individual atomic volumes^19^ and *V*_obs_ is the experimentally observed cell volume. Formation of the Ho_2_Ni_1–*x*_T_1+*x*_ phases is accompanied by 10–12% volume contraction, indicating a high thermodynamic stability of these compounds, which explains the high melting/decomposition temperatures (likely higher than 1650 °C, as noted above based on the DTA results).

**Table 3:** Stoichiometries of the Ho_2_Ni_1–*x*_T_1+*x*_ Phases with T = Si and Ge Determined from EDX and Rietveld Refinements, along with the Refined Lattice Parameters, Unit Cell Volumes, and Volume Contraction in Formation of the Compounds {Δ*V* % = [(*V*_obs_ – *V*_calc_)/*V*_calc_] × 100}

composition [atom %]					lattice parameters [Å]				
nominal (of the sample)	EDX (of the main phase)	chemical formula (EDX data)	chemical formula (from Rietveld)	composition in atom % (from Rietveld)	*a*	*b*	*c*	*V*_obs_ [Å^3^]	Δ*V* %
Ho_50_Ni_18.3_Si_31.7_	Ho_50(1)_Ni_18.5(1)_Si_31.5(1)_	Ho_2.01(4)_Ni_0.74(4)_Si_1.26(4)_	Ho_2_Ni_0.750(1)_Si_1.250(1)_	Ho_50_Ni_18.75(2)_Si_31.25(2)_	14.9136(1)	4.0986(1)	11.0688(1)	676.571(3)	–11.41
									
Ho_50_Ni_20_Si_30_	Ho_50(1)_Ni_20(1)_Si_30(1)_	Ho_2.02(4)_Ni_0.80(4)_Si_1.20(4)_	Ho_2_Ni_0.846(1)_Si_1.154(1)_	Ho_50_Ni_21.15(2)_Si_28.85(2)_	14.8988(1)	4.0996(1)	11.0606(1)	675.579(2)	–10.72
									
Ho_50_Ni_25_Si_25_	Ho_50(1)_Ni_22(1)_Si_28(1)_	Ho_2.00(4)_Ni_0.88(4)_Si_1.12(4)_	Ho_2_Ni_0.922(3)_Si_1.078(3)_	Ho_50_Ni_23.05(8)_Si_26.95(8)_	14.8798(1)	4.0995(1)	11.0550(1)	674.349(3)	–10.37
									
Ho_50_Ni_18.3_Ge_31.7_	Ho_51(1)_Ni_19(1)_Ge_30(1)_	Ho_2.04(4)_Ni_0.76(4)_Ge_1.20(4)_	Ho_2_Ni_0.799(1)_Ge_1.201(1)_	Ho_50_Ni_19.98(2)_Ge_30.02(2)_	15.0138(1)	4.1484(1)	11.0753(1)	689.804(9)	–12.16
									
Ho_50_Ni_20_Ge_30_	Ho_50(1)_Ni_20(1)_Ge_30(1)_	Ho_2.00(4)_Ni_0.80(4)_Ge_1.20(4)_	Ho_2_Ni_0.804(1)_Ge_1.196(1)_	Ho_50_Ni_20.11(1)_Ge_29.89(1)_	15.0054(1)	4.1483(1)	11.0719(1)	689.191(5)	–12.19
									
Ho_50_Ni_25_Ge_25_	Ho_50(1)_Ni_20(1)_Ge_30(1)_	Ho_2.00(4)_Ni_0.80(4)_Ge_1.20(4)_	Ho_2_Ni_0.810(1)_Ge_1.190(1)_	Ho_50_Ni_20.25(2)_Ge_29.75(2)_	14.9824(1)	4.1468(1)	11.0696(1)	687.748(7)	–12.31

Rietveld refinements confirm the bulk crystal structure determined using a small single crystal. Further, powder XRD data indicate that the two new orthorhombic phases exist over limited ranges of concentrations, that is, 0.094(1) ≤ *x* ≤ 0.250(1) (Δ*x* ≈ 0.16) for Ho_2_Ni_1–*x*_Si_1+*x*_ and 0.190(1) ≤ *x* ≤ 0.201(1) (Δ*x* ≈ 0.01) for Ho_2_Ni_1–*x*_Ge_1+*x*_. The much narrower and practically negligible solubility range of the germanide phase compared to that of the silicide phase is likely due to the larger atomic size of Ge compared to that of Si [atomic volumes (of elements in n.c.) of 22.64 and 20.02 Å^3^ for Ge and Si, respectively].^19^

**Figure 3:**
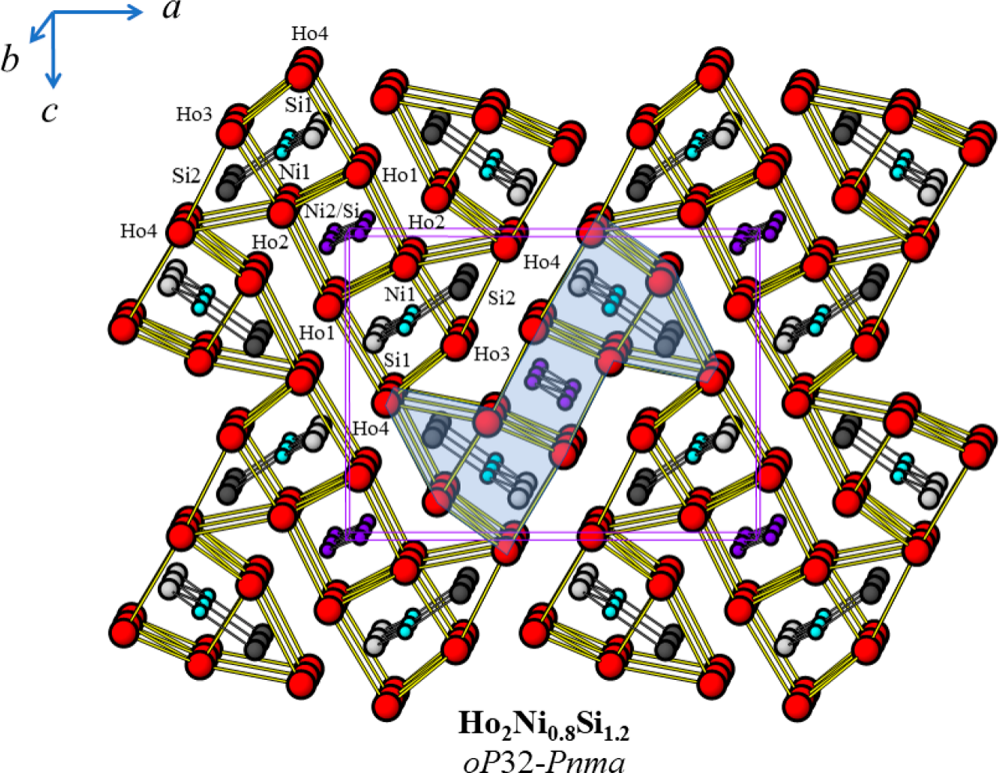
Crystal structure of Ho_2_Ni_0.8_Si_1.2_ [Zr_2_Ni_0.48_P-type; *oP*32; *Pnma* (No. 62)] viewed along the *b* axis. The Ho, Ni1, Ni2/Si, Si1, and Si2 atoms are represented as red, turquoise, blue, light-gray, and dark-gray spheres, respectively. The distinctive structural fragment built up of five differently oriented trigonal prisms is highlighted in transparent blue.

The Ho_2_Ni_1–*x*_T_1+*x*_ compounds belong to an extended family of rare-earth intermetallics, the crystal structures of which can be described by the packing of trigonal prisms formed by R atoms coordinating both M and T atoms (M and T are respectively a transition metal and a tetrel/*p*-block element). Here, these prisms are linked together by sharing square and triangular faces, forming a characteristic structural motif of interconnected columns, consisting of five trigonal prisms in a cross section, infinitely extending along the short *b*-axis direction (Figure 3). The three prisms in the middle of the cross section are arranged so that the (pseudo)-3-fold prism axes are parallel to the *ac* plane, while the two prisms located at the ends are oriented with their axes along the *b* axis (Figure 3).

**Figure 4:**
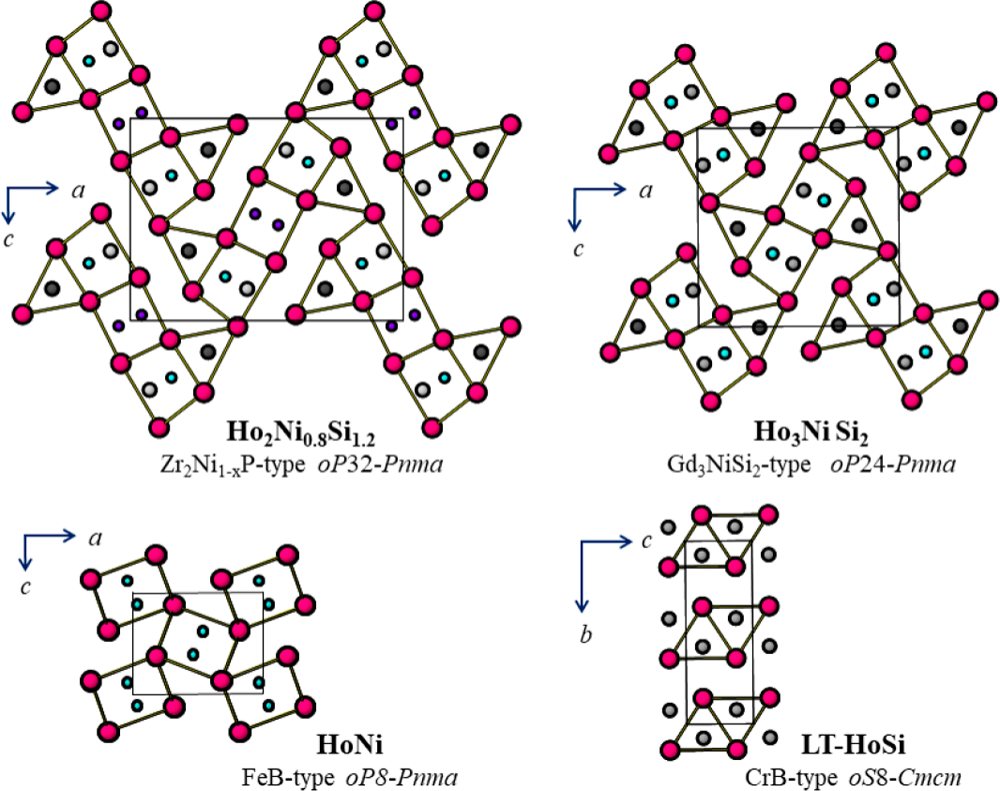
Upper part: Comparison between the two structures of Ho_2_Ni_0.8_Si_1.2_ (Zr_2_Ni_0.48_P type; *oP*32; *Pnma*) and Ho_3_NiSi_2_ (Gd_3_NiSi_2_ type; *oP*24; *Pnma*), showing characteristic trigonal-prismatic coordination and similar orientation and concatenation of the respective structural motifs. Lower part: Linking of the trigonal prisms in the binary compounds HoNi (FeB type) and HoSi (low-temperature CrB type).

The trigonal prisms may be distorted depending on the kind of atom hosted inside them; i.e., the prism edges are longer (shorter) when the prism coordinates the larger (smaller) Si (Ni) atoms. These distortions are similar to those observed in the binary HoNi [FeB-type, *oP*8; *Pnma* (No. 62)] and HoSi [CrB-type; *oS*8; *Cmcm* (No. 63) as the low-T form; FeB-type as the high-T form] compounds;^8,9^ both structure types are based on the trigonal-prismatic coordination of the Ni and T atoms (Figure 4). The ordering of the Ni and T atoms in Ho_2_Ni_1–*x*_T_1+*x*_ is such that (i) the number of heteroatomic bonds (Ni–T) is maximized and (ii) the number of homoatomic T–T contacts is minimized (limited to those pertaining to the mixed site Ni2/T, where Ni and T are almost equally distributed). This result is quite interesting because it suggests a strong bonding interaction between the Ni and T atoms, and because a stoichiometric T/Ni ratio of about 1.5 should instead favor the formation of T–T bonds. In Figure 4, the structure of Ho_2_Ni_0.769(5)_Si_1.231(5)_ is also compared with that of Ho_3_NiSi_2_ (Gd_3_NiSi_2_-type). Both compounds show a similar arrangement of structural fragments, with characteristic trigonal-prismatic coordination and a similar orientation of the respective structural motifs. All of the Ho atoms show the same coordination number CN = 17, and their coordination polyhedra are very similar, corresponding to irregular pentagonal prisms capped on all of the faces.

We also found that this new R_2_Ni_1–*x*_T_1+*x*_ phase forms for R = Tb with Ge and for R = Y, Dy, and Ho with both Si and Ge;^20^ these phases will be the subject of a forthcoming report. Notably, the R_2_Ni_1–*x*_T_1+*x*_ compounds represent the first known examples of ternary R-based phases adopting the structure of the Zr_2_Ni_1–*x*_P prototype.^6−9^ Even though not directly related to the subject of this work, our EDX and powder XRD data have also proven formation of the compound Ho_3_NiGe_2_, crystallizing in an orthorhombic cell of the Gd_3_NiSi_2_-type [*oP*24; *Pnma* (No. 62)]^21^ with lattice parameters *a* = 11.2922(1) Å, *b* = 4.1542(1) Å, and *c* = 11.1410(1) Å.

### 3.2 Physical Properties of the Ho_2_Ni_0.8_T_1.2_ Compounds

#### 3.2.1 Magnetic Properties

**Figure 5:**
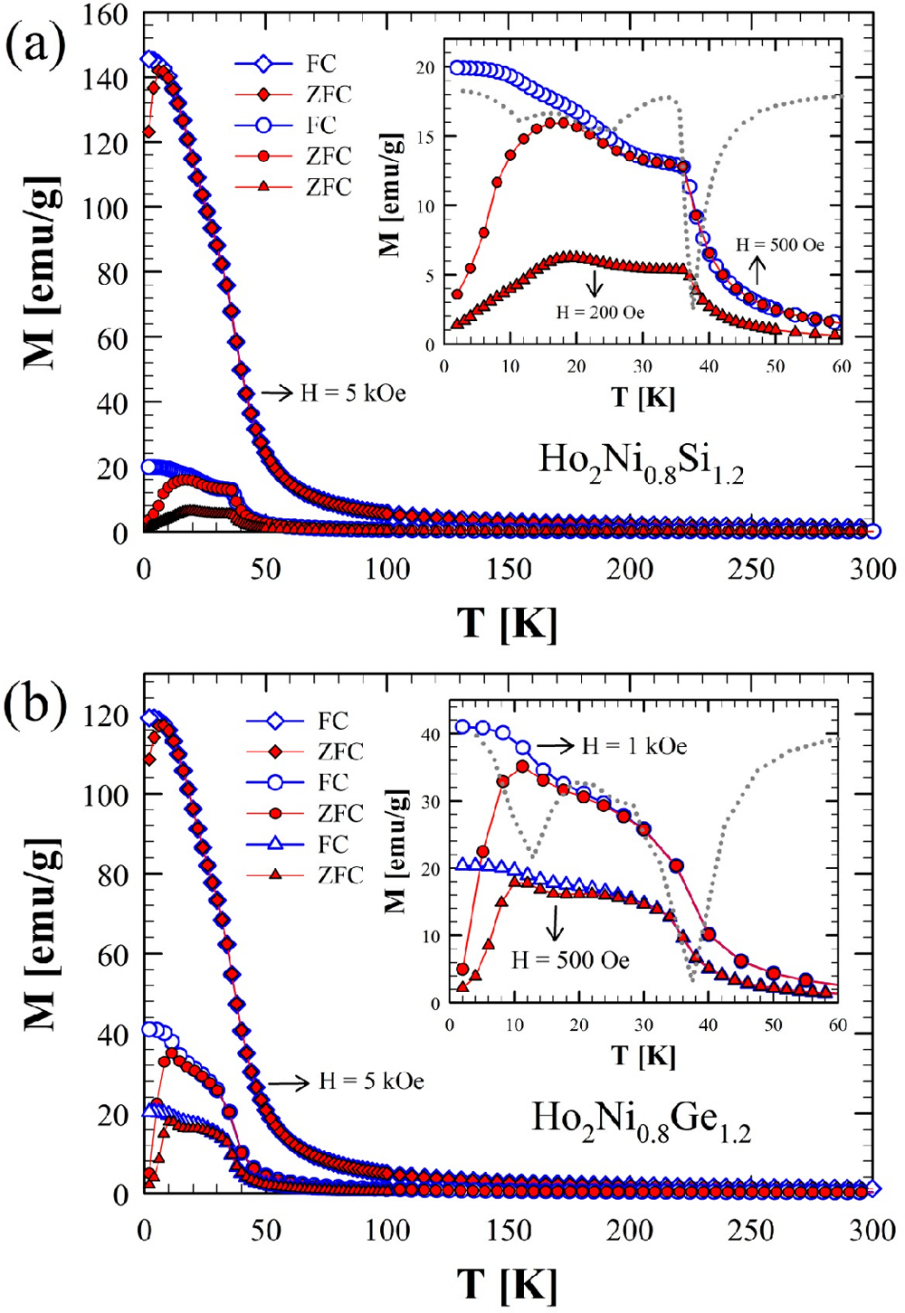
dc magnetic susceptibility versus temperature measured between 2 and 300 K at 200 Oe, 500 Oe, and 5 kOe for Ho_2_Ni_0.8_Si_1.2_ (a) and at 500 Oe, 1 kOe, and 5 kOe for Ho_2_Ni_0.8_Ge_1.2_ (b). The insets show an enlarged view of the ZFC and FC magnetization data (0–60 K) measured at 200 and 500 Oe for Ho_2_Ni_0.8_Si_1.2_ (inset in part a) and at 500 Oe and 1 kOe for Ho_2_Ni_0.8_Ge_1.2_ (inset in part b); the dotted lines in both insets represent the first derivative of the magnetization data for the data measured at the higher field [500 Oe for Ho_2_Ni_0.8_Si_1.2_ (a) and 1 kOe for Ho_2_Ni_0.8_Ge_1.2_ (b)].

The temperature dependencies of the magnetization, *M*(*T*), have been measured between 2 and 300 K in applied magnetic fields of 200 Oe, 500 Oe, and 5 kOe for Ho_2_Ni_0.8_Si_1.2_ (Figure 5a) and of 500 Oe, 1 kOe, and 5 kOe for Ho_2_Ni_0.8_Ge_1.2_ (Figure 5b).

The magnetic behaviors of these two compounds are similar. The *M*(*T*) data reveal main transitions that are either ferrimagnetic (FIM) or ferromagnetic (FM) in nature, occurring at *T*_C_ = 38 K for the silicide and *T*_C_ = 37 K for the germanide. These transition temperatures are determined from the minima of the first derivatives of the magnetization with respect to the temperature (d*M*/d*T*; FC data at 500 Oe; see the insets of parts a and b of Figure 5, respectively). The derivatives plotted as a function of the temperature also reveal weak and broad additional minima at about 25 and 11 K for Ho_2_Ni_0.8_Si_1.2_ and at 13 K for Ho_2_Ni_0.8_Ge_1.2_. Considering the phase purity of both materials (Tables S4 and S7), it is reasonable to assume that the additional low-temperature anomalies are intrinsic to these compounds. Thermomagnetic irreversibilities present between the ZFC and FC data below about 10–20 K are consistent with the nonzero hysteresis developing at the lowest temperature.

**Figure 6:**
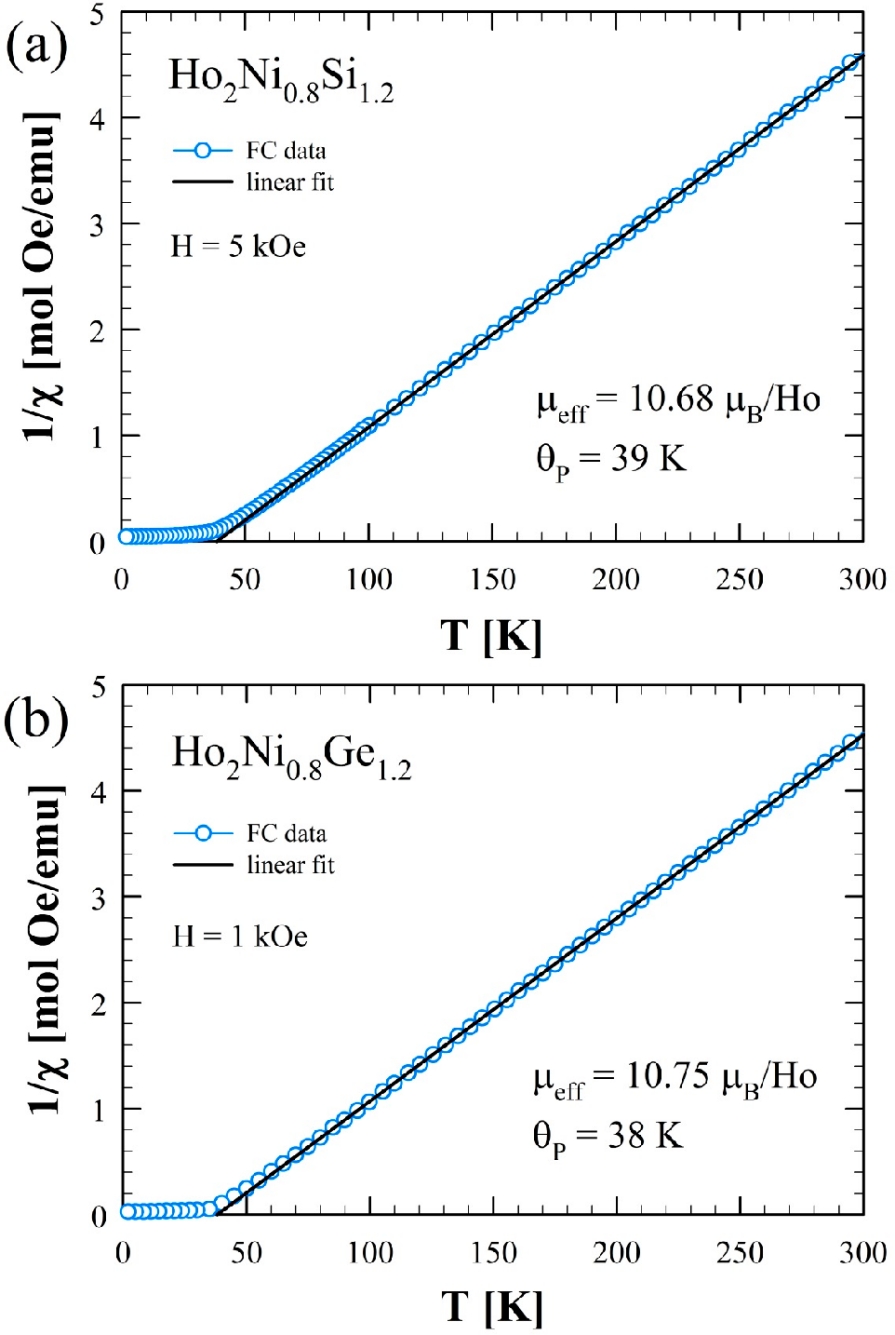
Plots of the inverse magnetic susceptibility measured between 2 and 300 K and at 5 kOe for Ho_2_Ni_0.8_Si_1.2_ (a) and 1 kOe for Ho_2_Ni_0.8_Ge_1.2_ (b). The straight lines are the fits to the Curie–Weiss law.

The inverse magnetic susceptibility, 1/χ = *H*/*M*, is shown in parts a and b of Figure 6 for the two compounds, respectively. The data follow the Curie–Weiss law χ(*T*) = *C*/(*T* – θ_P_) (with *C* being the Curie constant) above 100 K for the silicide and above 75 K for the germanide. The least-squares fits to the data in the paramagnetic region give an effective magnetic moment, *p*_eff_, of 10.68 μ_B_ for Ho_2_Ni_0.8_Si_1.2_ and 10.75 μ_B_ for Ho_2_Ni_0.8_Ge_1.2_. Both values are very close to the Hund’s rule derived theoretical value of 10.61 μ_B_ for the Ho^3+^ ion,^23^ indicating that the Ni magnetic moment is quenched, as is common for many other rare-earth compounds containing Ni.^24,25^ The positive values of the Weiss temperatures, θ_P_, of 39 and 38 K respectively for the silicide and germanide are commensurate with either the FM or FIM ground states.

**Figure 7:**
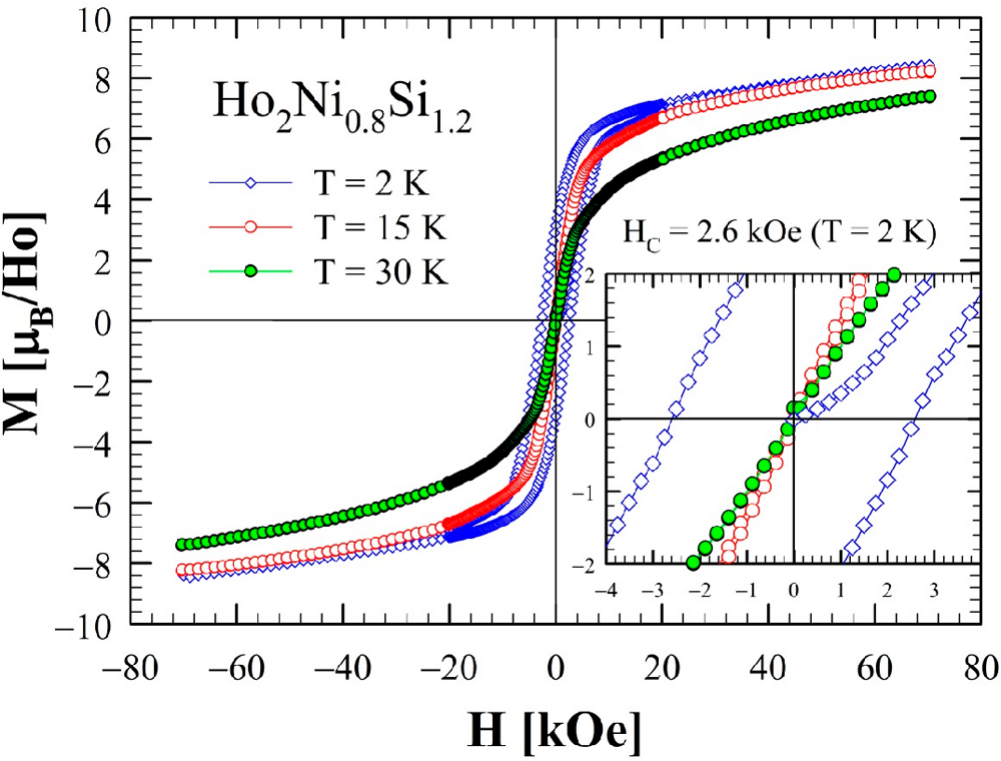
Isothermal magnetization of Ho_2_Ni_0.8_Si_1.2_ measured at 2, 15, and 30 K in the range of ±70 kOe. The inset shows an enlarged view of the data between −4 and +4 kOe (*H*_C_ = 2.6 kOe at 2 K).

**Figure 8:**
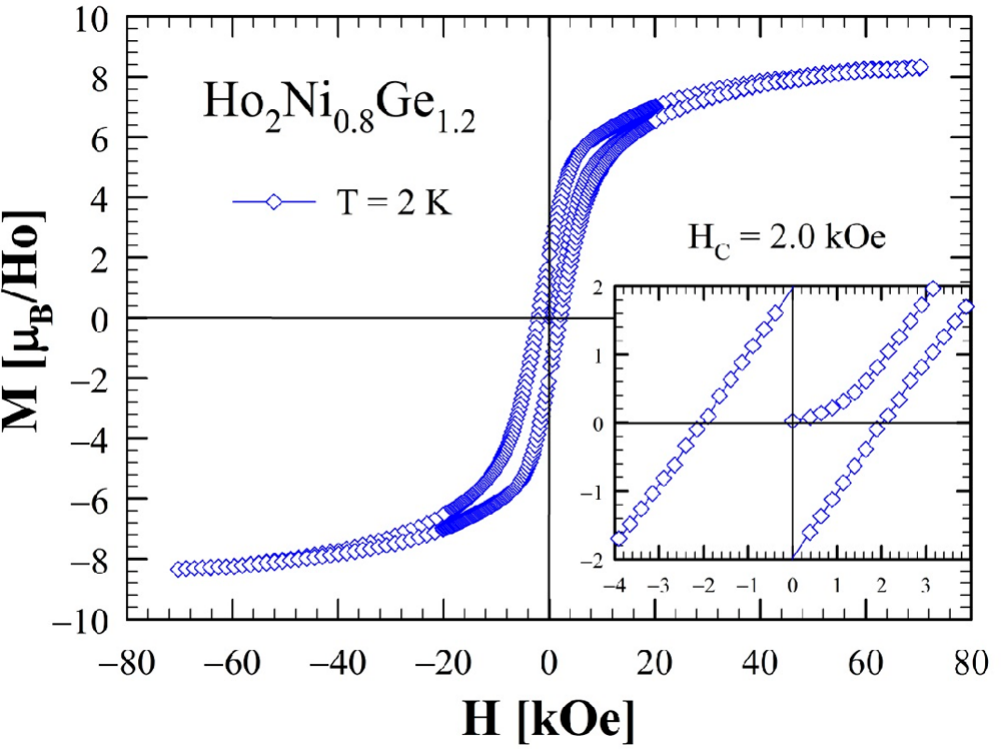
Isothermal magnetization of Ho_2_Ni_0.8_Ge_1.2_ measured at 2 K in the range of ±70 kOe. The inset shows an enlarged view of the data between −4 and +4 kOe (*H*_C_ = 2.0 kOe at 2 K).

The isothermal magnetization, *M*(*H*), measured at *T* = 2 K (both compounds) and 15 and 30 K for the silicide in magnetic fields up to 70 kOe, is shown in Figures 7 and 8.

The measurements at *T* = 2 K show noticeable hysteresis, which is typical of FM/FIM materials formed by lanthanides with nonzero single-ion anisotropies. The *M*(*H*) data do not reach saturation even at the highest applied magnetic field of 70 kOe, approaching 8.33 μ_B_/Ho atom, a value lower than the expected 10 μ_B_/Ho if all Ho magnetic moments would align ferromagnetically, possibly indicating antiferromagnetic (AFM) contributions rather than a simple collinear FM ordering. A comparison between the isothermal magnetization of the two compounds, for data measured at 2 K, is shown in Figure S2; a very similar behavior is observed, with the coercive field slightly larger for the silicide (*H*_C_ at 2 K is 2.6 and 2.0 kOe for Ho_2_Ni_0.8_Si_1.2_ and Ho_2_Ni_0.8_Ge_1.2_, respectively).

#### 3.2.2 Heat Capacity

**Figure 9:**
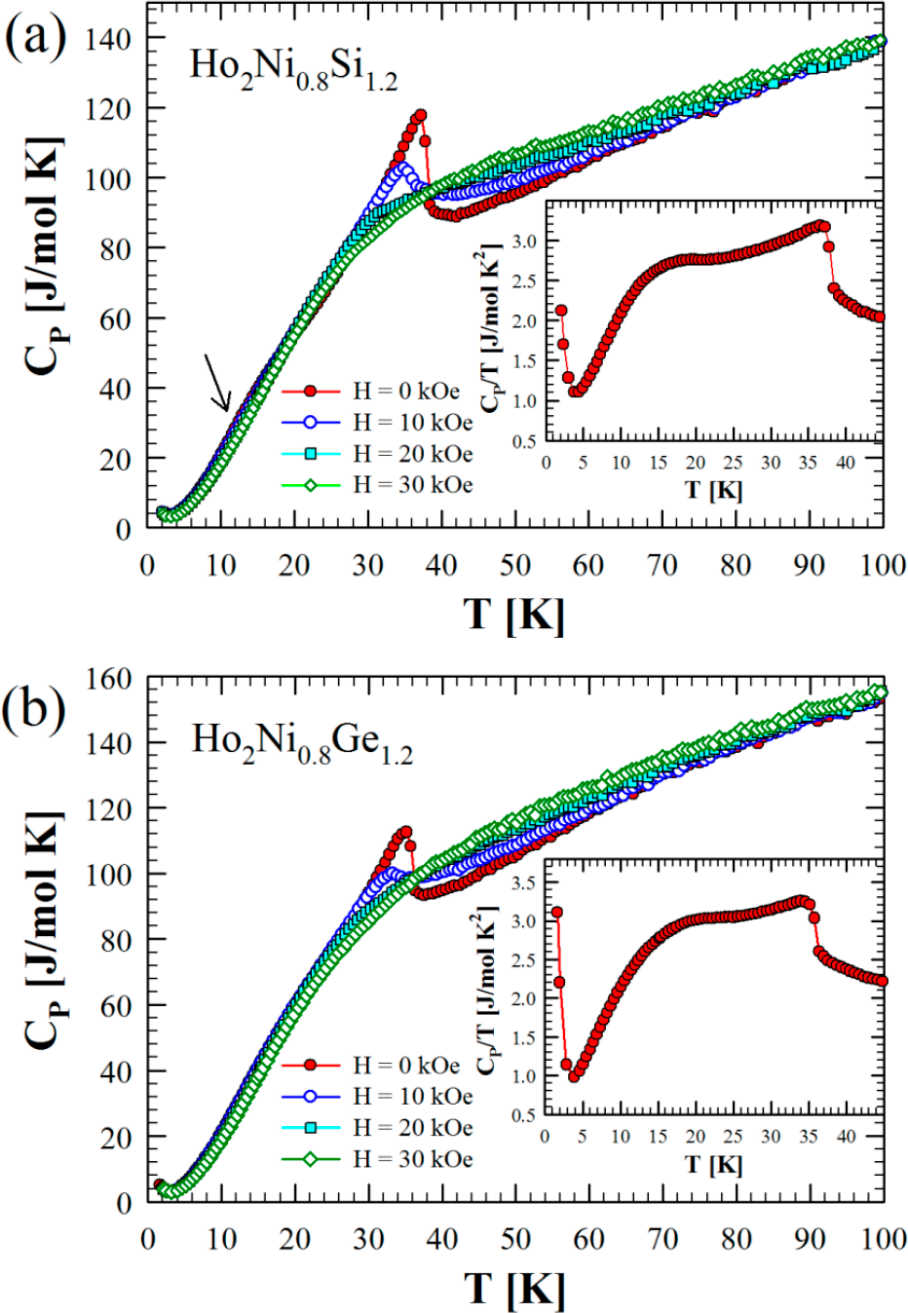
Heat capacity data versus temperature in the range 2–100 K measured in zero and applied magnetic fields of 10, 20, and 30 kOe for Ho_2_Ni_0.8_Si_1.2_ (a) and Ho_2_Ni_0.8_Ge_1.2_ (b). The insets show the zero-field *C*_P_/*T* data versus *T* between 2 and 45 K.

The heat capacity has been measured for both compounds between 2 and 100 K in zero and applied magnetic fields of 10, 20, and 30 kOe (Figure 9a,b).

**Figure 10:**
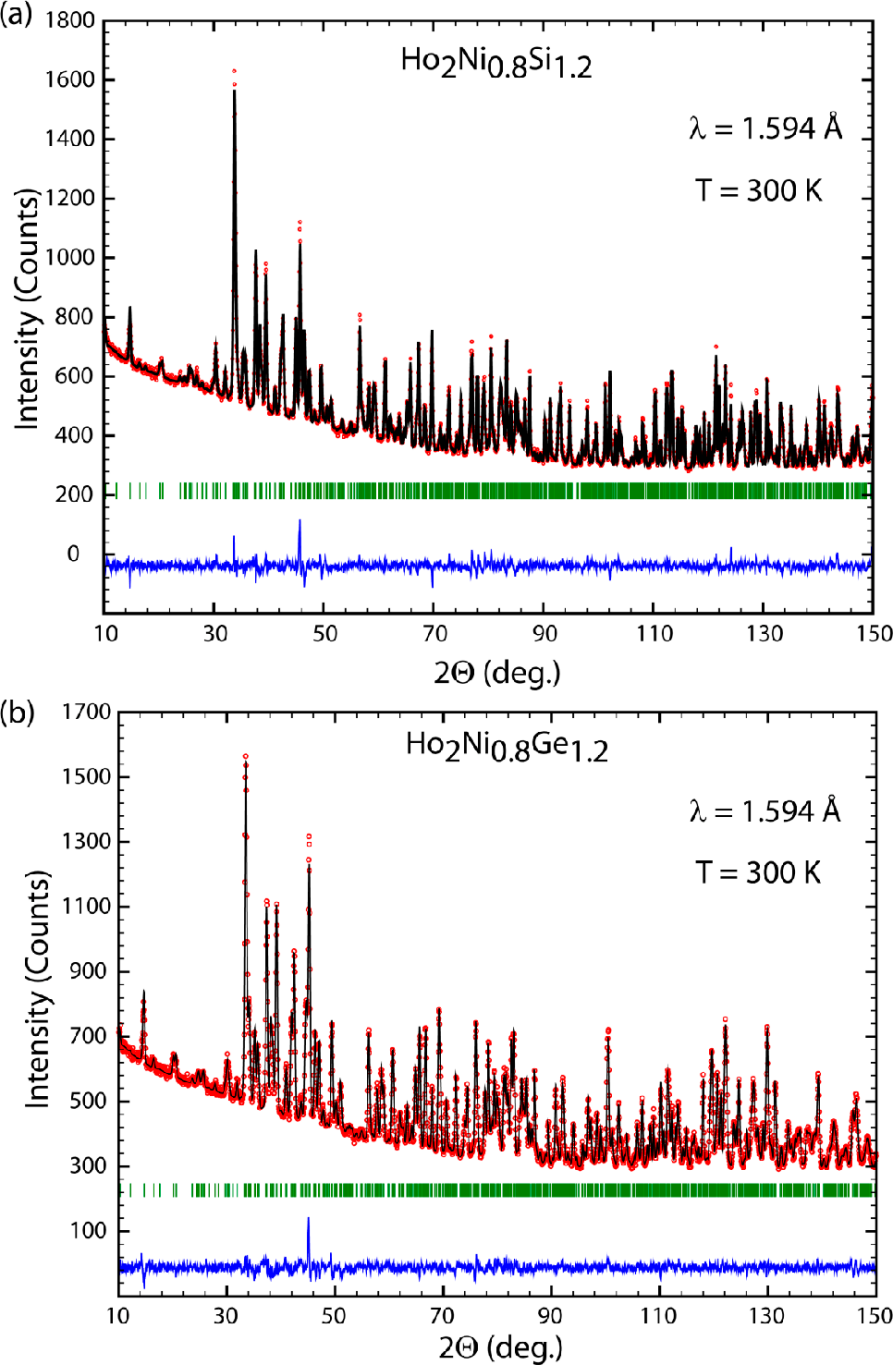
High-resolution neutron powder diffractograms for the Ho_2_Ni_0.8_Si_1.2_ (a) and Ho_2_Ni_0.8_Ge_1.2_ (b) compounds.

The data show the main λ-type peaks in agreement with the global magnetic ordering transition temperatures determined from the *M*(*T*) data, indicating that these phase changes are second-order. Plotting *C*_P_/*T* versus *T* of the zero-field data (insets of Figure 10) clearly reveals additional broad anomalies, approximately matching the weak anomalies seen in the *M*(*T*) data. Rapid upturns observed below ∼4 K in both compounds reflect the hyperfine field contributions commonly observed in other Ho-based intermetallics.^26^ Under the applied magnetic field, the main peaks initially move to lower temperatures, suggesting at least some degree of antiparallel coupling between the Ho magnetic moments in both compounds. This supports the idea mentioned above that the low value of the magnetization at 70 kOe and its nonsaturation might be caused by AFM contributions. Broad anomalies are also observed at about 12 K for both compounds, likely indicating spin reordering under a magnetic field.

### 3.3 Magnetic Structure

High-resolution powder neutron diffraction data confirm the orthorhombic crystal structure of both the Ho_2_Ni_0.8_Si_1.2_ and Ho_2_Ni_0.8_Ge_1.2_ compounds as well as the mixed Ni/Si occupation of only one of the eight 4*c* Wyckoff sites (Figure 10a,b), with the final refined stoichiometries being about Ho_2_Ni_0.78(1)_Si_1.22(1)_ and Ho_2_Ni_0.76(2)_Ge_1.24(2)_. No sign of the presence of any visible amounts of impurity phases is found.

**Figure 11:**
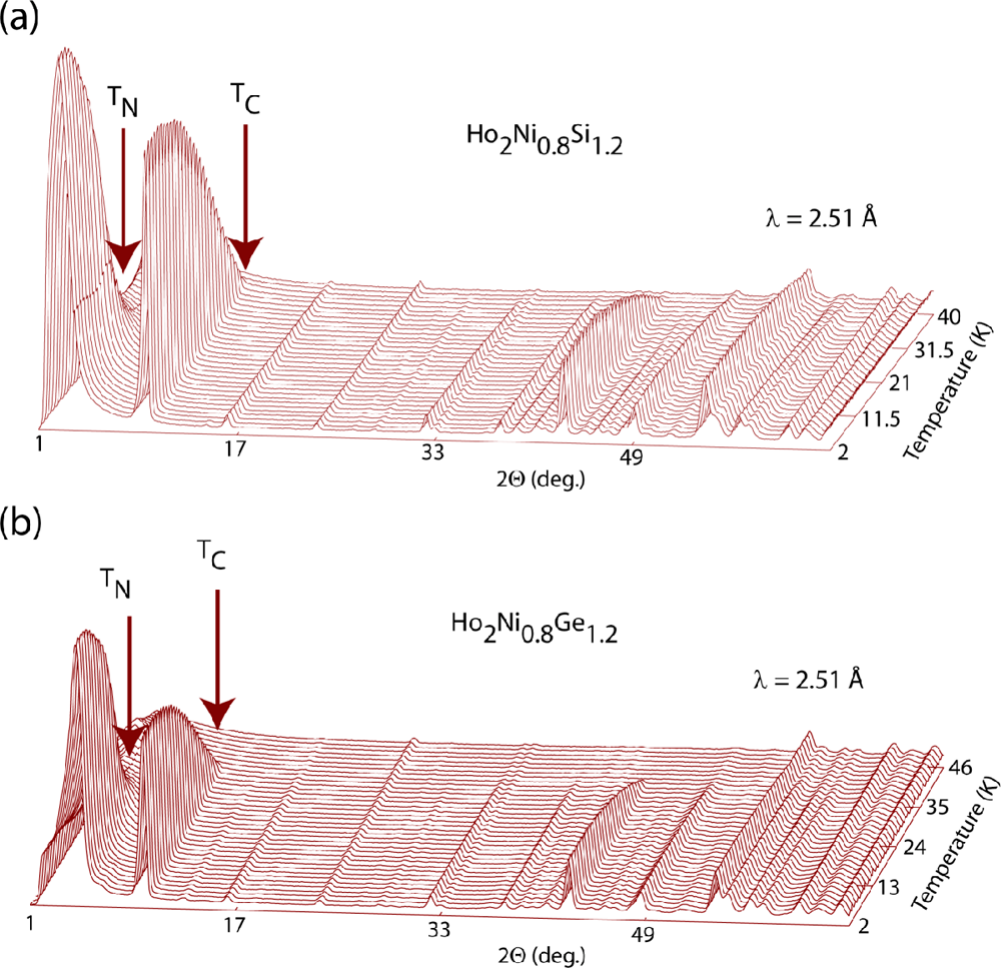
Neutron thermodiffractograms for the Ho_2_Ni_0.8_Si_1.2_ (a) and Ho_2_Ni_0.8_Ge_1.2_ (b) compounds.

The thermodiffractograms of the two compounds as measured using high-intensity powder neutron diffraction are shown in Figure 11a,b. A first magnetic transition manifests as the appearance of new Bragg peaks and the increase of the intensity of some nuclear Bragg peaks at *T*_C_ = 38 and 37 K for the silicide and germanide, respectively.

All magnetic Bragg peaks can be indexed using the program *K-search*, which is part of the *FullProf* suite of programs^12^ with a magnetic propagation vector κ_1_ = [0, 0, 0]. Figure S3 shows the integrated intensity of the strongest among all of the magnetic peaks [i.e., the (1, 0, 0) magnetic peak] of this κ_1_ phase (the first magnetic phase appearing upon cooling), as a function of the temperature, for Ho_2_Ni_0.8_Ge_1.2_ (Figure S3a) and Ho_2_Ni_0.8_Si_1.2_ (Figure S3b). A second magnetic transition seems to take place at about *T*_N_ = 24 K in Ho_2_Ni_0.8_Si_1.2_ and at about 22 K in Ho_2_Ni_0.8_Ge_1.2_, where a single new magnetic Bragg peak at very low 2θ values appears in both compounds. We will first discuss the magnetic structure present below *T*_C_ before we deal with the situation below *T*_N_. Magnetic symmetry analysis using the program *BASIREPS*(17,18) determined the allowed irreducible representations (IRs) and their basis vectors (BVs) for κ_1_ = [0, 0, 0] for the Wyckoff position 4*c* in the space group *Pnma* (Table S9).

**Figure 12:**
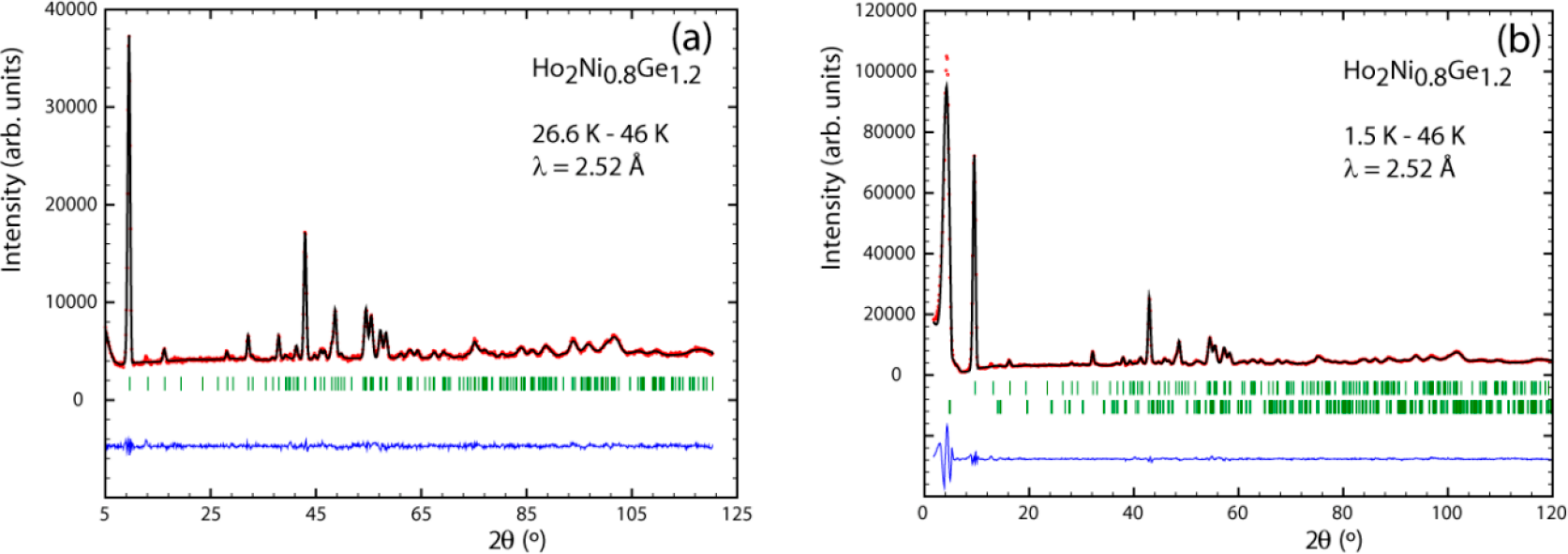
Neutron intensity difference patterns at 26.6–46 K (a) and 1.5–46 K (b) for Ho_2_Ni_0.8_Ge_1.2_.

Among the eight allowed IRs, only one having a FM BV along the unit cell *a* direction and an AFM coupling (BV) in the *c* direction (IR7; Table S9) allows refinement of the diffraction data below *T*_C_. In order to have an increased sensitivity to the magnetic diffraction intensity, we refined difference data sets created by subtracting the purely nuclear intensity recorded in the paramagnetic region above *T*_C_. Figures 12a and 14 display the refinement of the difference patterns created by subtracting the 46 K data from the 26.6 K data for the germanide and the 40 K data from the 24.5 K data for the silicide.

**Figure 13:**
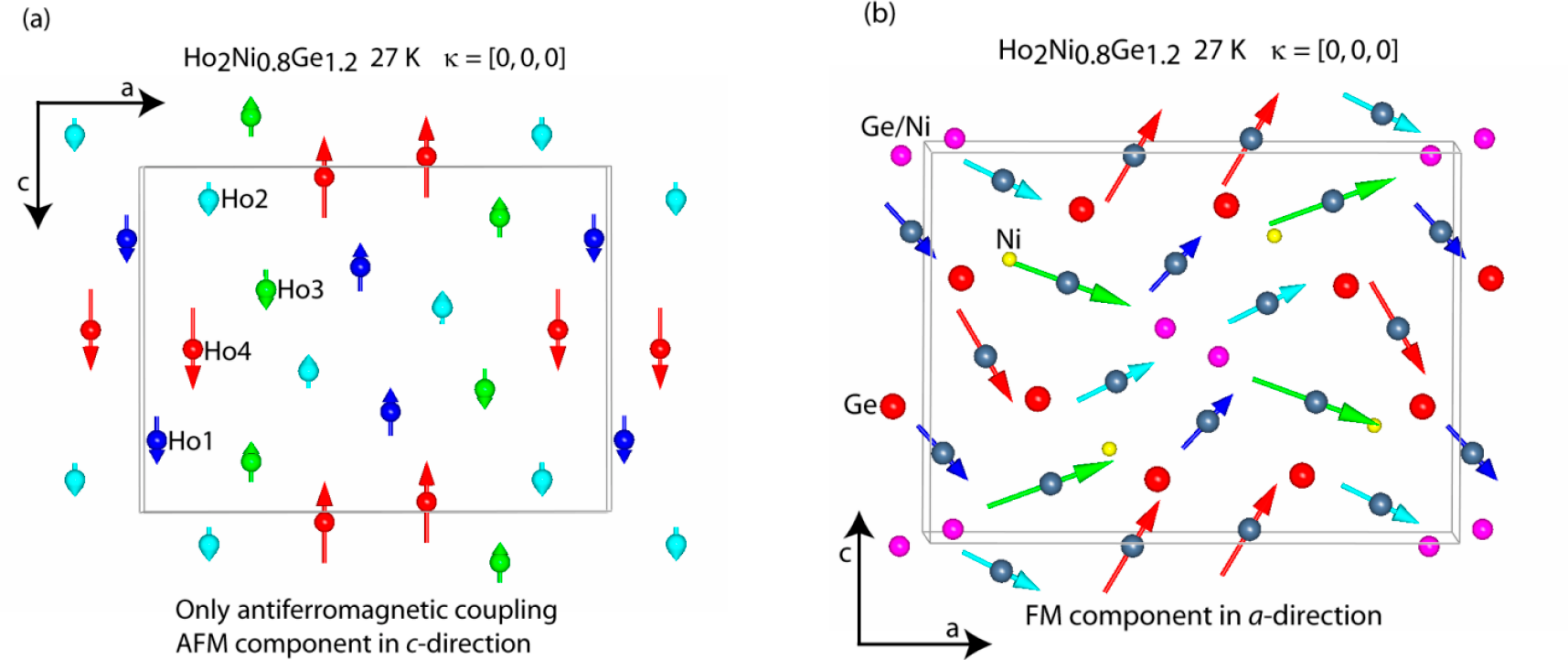
Magnetic structure of Ho_2_Ni_0.8_Ge_1.2_ at 27 K: AFM coupling in the *c* direction (a); FM component in the *a* direction (b).

**Figure 14:**
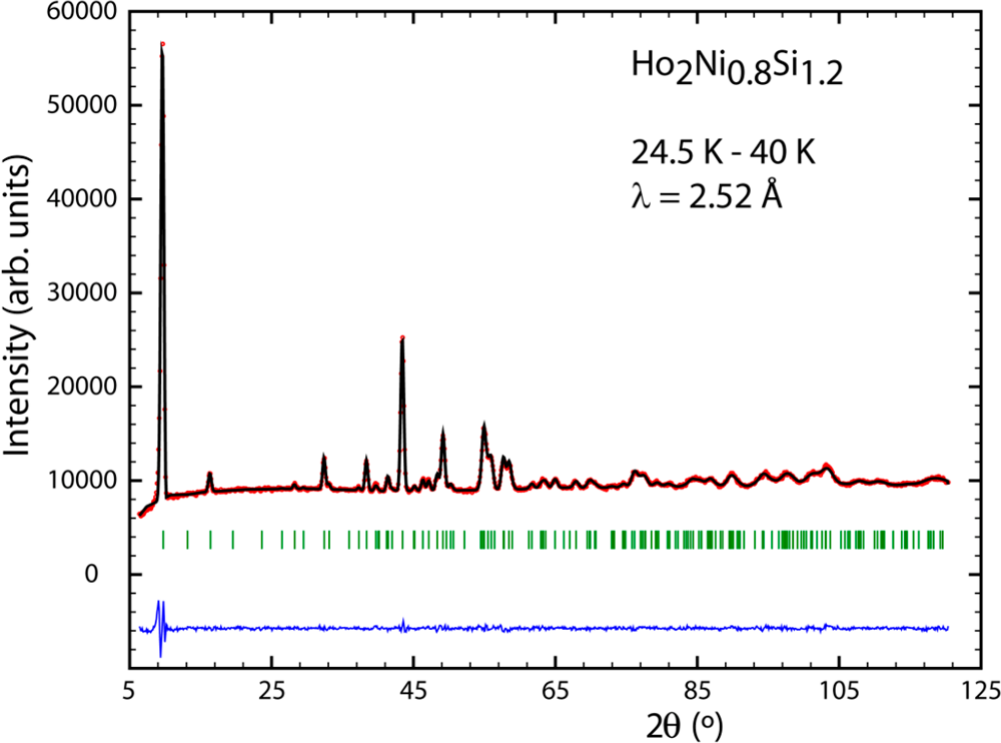
Neutron intensity difference pattern at 24.5–40 K for Ho_2_Ni_0.8_Si_1.2_.

Both compounds possess the same magnetic structure, which is displayed in an exemplary way for Ho_2_Ni_0.8_Ge_1.2_ in Figure 13b. The magnetic unit cell is the same as the crystalline cell. The AFM component consists of FM stripes along the *c* direction of Ho1, Ho2, and Ho4 that are antiferromagnetically aligned along the *a* direction, separated by an AFM strip of Ho3 spins, which extends as well along the *c*-axis direction (Figure 13a).

As the FM component along the *a* direction is added, the resulting magnetic structure is obtained, which represents a canted magnetic structure. Looking at the temperature dependence of the different magnetic reflections (Figure 11), they show a similar monotonic behavior indicating that the four Ho magnetic moments not only follow the same magnetic propagation vector but also order at the same temperature. This is equally true for both compounds. Even though all Ho sites occupy the same Wyckoff position (4*c*), this is not obvious, as has been shown, for example, in the intermetallics Ho_5_Ni_2_In_4_ or Ho_11_Ni_4_In_9_, where different magnetic propagation vectors and different temperature dependencies have been found even for Ho ions occupying the same Wyckoff site.^25^Table 4 reports for Ho_2_Ni_0.8_Ge_1.2_ at 26.6 K and for Ho_2_Ni_0.8_Si_1.2_ at 24.5 K the values of the AFM component along the *c* direction, of the FM component along the *a* direction, and of the resulting total magnetic moment for all of the Ho atoms.

**Table 4:** Magnetic Moments and Their Components along the *a* and *c* Axes for Ho_2_Ni_0.8_Si_1.2_ and Ho_2_Ni_0.8_Ge_1.2_ above *T*_N_

	Ho_2_Ni_0.8_Si_1.2_ (*T* = 24.5 K)			Ho_2_Ni_0.8_Ge_1.2_ (*T* = 26.6 K)		
R	FM [μ_B_]∥*a*	AFM [μ_B_]∥*c*	μ_Ho_ [μ_B_]	FM [μ_B_]∥*a*	AFM [μ_B_]∥*c*	μ_Ho_ [μ_B_]
Ho1	2.9(1)	3.2(1)	4.3(1)	2.8(1)	3.1(1)	4.2(1)
Ho2	5.2(1)	2.6(1)	5.8(1)	4.4(1)	2.2(1)	4.9(1)
Ho3	7.2(1)	2.7(1)	7.7(1)	7.1(1)	2.6(1)	7.6(1)
Ho4	3.3(1)	–5.8(1)	6.7(1)	3.1(1)	–5.2(1)	6.15(1)

As was already indicated above, the appearance of a very strong additional magnetic Bragg peak at very low 2θ values indicates a change in the magnetic structure and can be looked upon as a second magnetic transition defining a Néel temperature *T*_N_. The closeness of the magnetic transition temperature *T*_N_ ∼ 24 K of Ho_5_Si_3_^22^—a possible impurity phase—to the here determined *T*_N_ = 24 K for the silicide compound and a certain similitude of the main magnetic Bragg peak could question the origin of this second transition as coming from the Ho_2_Ni_0.8_Si_1.2_ sample. The absence of any visible impurity phase in the high-resolution and high-intensity neutron diffraction data and the strong intensity of the additional low-angle magnetic peak exclude, however, this possibility. The same arguments speak against the origin of the low-angle magnetic peak in Ho_2_Ni_0.8_Ge_1.2_ as coming from a hypothetical Ho_5_Ge_3_ impurity; furthermore, in this case the transition temperatures are significantly different because *T*_N_ = 27 K for Ho_5_Ge_3_^22^ and *T*_N_ = 22 K for Ho_2_Ni_0.8_Ge_1.2_.

**Figure 15:**
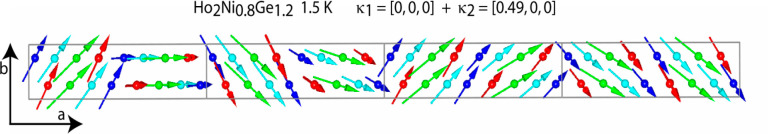
Magnetic structure of Ho_2_Ni_0.8_Ge_1.2_ at 1.5 K resulting from the superposition of the κ_1_- and κ_2_-type magnetic orders. The figure is drawn with κ_2_ = [0.45, 0, 0] to emphasize changes between neighboring unit cells.

The fact that only one broad new magnetic peak appears makes the determination of a magnetic propagation vector ambiguous, because an unlimited number of incommensurate values can be found that reproduce the position of the peak. Limiting oneself to magnetic propagation vectors having only one component and using the information on the absence of further new magnetic peaks, a simple solution can be found where κ_2_ = [≈0.36, 0, 0] for Ho_2_Ni_0.8_Si_1.2_ and κ_2_ = [0.49, 0, 0] for Ho_2_Ni_0.8_Ge_1.2_. Magnetic symmetry analysis proposes four IRs, with two representing sine waves and two others representing cycloidal types of magnetic structures. Only one sine-wave model is able to reproduce the magnetic peak and the absence of any further magnetic peaks with reasonable magnetic moment values (Figure 12b). This sine-wave structure sees the magnetic component pointing along the unit cell *b* direction with a maximum amplitude of 5.0(1) μ_B_ at 1.5 K for Ho_2_Ni_0.8_Ge_1.2_. Here, all Ho sites were constrained to have the same amplitude and the same phase. The superposition of this κ_2_ = [κ_*x*_, 0, 0] modulation with the κ_1_ = [0, 0, 0] order leads to a magnetic structure sketched in Figure 15.

Table 5 lists the FM and AFM components of the κ_1_ order and of the sine wave at 1.5 K, together with the resulting total magnetic moments of each site. The refinement assumed hereby that both magnetic couplings extend over the whole sample volume. A phase segregation scenario where one part of the sample volume follows κ_1_ and the second part κ_2_ cannot be taken into consideration as a solution because the total moment values would be higher than the free ion value of Ho^3+^, which is μ_Ho^3+^_ = 10.61 μ_B_. The fact that the temperature dependence of the magnetic peaks created at *T*_C_ do not show any change at *T*_N_ (Figure 12a,b) speaks as well in favor of the additional magnetic coupling appearing at *T*_N_ to act on top of the already existing magnetic order.

**Table 5:** Magnetic Moments and Their Components along the *a* and *c* Axes for Ho_2_Ni_0.8_Ge_1.2_ and Ho_2_Ni_0.8_Si_1.2_ at 1.5 K within the κ_1_-Type Structure and Total Magnetic Moment Values for Ho_2_Ni_0.8_Ge_1.2_ Resulting from Superposition with the κ_2_-Type Order

	Ho_2_Ni_0.8_Ge_1.2_ (*T* = 1.5 K)			
	κ_1_ = [0, 0, 0]		κ_2_ = [0.49, 0, 0]	κ_1_ + κ_2_
R	FM [μ_B_]∥*a*	AFM [μ_B_]∥*c*	AFM [μ_B_]∥*b*	μ_Ho_ [μ_B_]
Ho1	4.2(1)	5.1(1)	maximum amplitude for all: 5.0(5)	6.6–8.3
Ho2	5.6(1)	3.0(1)		6.4–8.1
Ho3	7.8(1)	3.0(1)		8.3–9.7
Ho4	3.4(1)	–7.2(1)		8.0–9.4

	Ho_2_Ni_0.8_Si_1.2_ (*T* = 1.5 K)		
R	FM [μ_B_]∥*a*	AFM [μ_B_]∥*c*	μ_Ho_ [μ_B_]
Ho1	3.5(1)	4.1(1)	5.8(1)
Ho2	6.1(1)	2.8(1)	6.7(1)
Ho3	7.7(1)	2.8(1)	8.2(1)
Ho4	3.6(1)	–7.6(1)	8.4(1)

Because of the closeness of the first magnetic peak to the direct beam, it was not possible to attempt a refinement of the silicide data at 1.5 K including this second magnetic phase. However, it can be assumed that this second magnetic order is similar to that of the germanide because it shows a very similar temperature dependence and the same absence of additional new magnetic peaks. Values of the refined magnetic moments corresponding to the κ_1_ phase of Ho_2_Ni_0.8_Si_1.2_ at 1.5 K are included in Table 5.

Comparing the results of neutron diffraction with those of the heat capacity and magnetic data, the question remains, why is the second transition at *T*_N_ not clearly seen in the *C*_P_ and magnetic data of the germanide and only very faintly seen in the data of the silicide. At least for Ho_2_Ni_0.8_Ge_1.2_, a hint is given by the temperature dependence of the peak width and peak position of the magnetic peak at very low angles, which has been used to define *T*_N_. Figure S4a–c shows that, as the temperature approaches the assumed value of *T*_N_ ∼ 24 K, the peak position drifts to even lower angles, while the peak width strongly increases at the same time. This could indicate that the underlying AFM coupling is already present at higher temperatures but not developing sufficient long-range order to create a sharp peak visible in the diffraction data. This interpretation assumes therefore that at *T*_C_ (where the κ_1_ order sets in) short-range order of the κ_2_ type appears as well already. No further clear peak in the *C*_P_ data is therefore created at *T*_N_ because only the correlation length of the coupling and the value κ_*x*_ of the magnetic propagation vector κ_2_ are changing. Figure S4 shows, furthermore, that a small anomaly is visible at around 13–14 K in the temperature dependence of the intensity of the strong low-angle peak of the κ_2_ phase as well as in its full width at half-maximum and its position. It indicates a small change in the details of this incommensurate magnetic structure and can be related to the small anomaly seen in the magnetization data at 13 K.

## 4 Summary

The crystal structure, magnetic properties, and magnetic structures of two new rare-earth-based intermetallic compounds, Ho_2_Ni_0.8_T_1.2_ with T= Si and Ge, are reported. They correspond to the unidentified phase R_50_Ni_20_T_30_, namely, “Ho_5_Ni_2_T_3_”, earlier reported to form in the ternary systems Dy–Ni–T and Ho–Ni–T and crystallize with a filled version of the orthorhombic unit cell of the Zr_2_Ni_1–*x*_P type [space group *Pnma* (No. 62); Pearson symbol *oP*32–y)]. While this prototype presents a vacancy of *x* = 0.52, which translates into a resulting stoichiometry of Zr_2_Ni_0.48_P, the stoichiometry of the two Ho compounds studied here is centered on Ho_2_Ni_0.8_T_1.2_ with a very narrow solid solubility range for the silicide, while the germanide turns out to be a line phase. In addition to R = Dy and Ho, R_2_Ni_0.8_T_1.2_ compounds are also formed for R = Y and Tb; attempts to prepare the homologous Gd-based Gd_2_Ni_0.8_T_1.2_ failed. The R_2_Ni_0.8_T_1.2_ compounds constitute the first example of an R-based compound crystallizing with the Zr_2_Ni_1–*x*_P type, as well as the first case of an intermetallic phase adopting this ternary structural prototype.

FIM- or FM-type ordering, at 38 K for Ho_2_Ni_0.8_Si_1.2_ and 37 K for Ho_2_Ni_0.8_Ge_1.2_, is revealed by the magnetization data; this main transition is then followed by subsequent magnetic orderings at lower temperatures. The susceptibility data in the paramagnetic region give effective moments, μ_eff_, of 10.68 and 10.75 μ_B_ for the silicide and germanide, respectively; both values are very close to the theoretical value of 10.61 μ_B_. Neutron diffraction shows the existence of two magnetic propagation vectors in both compounds. First transitions at *T*_C_ = 38 K for the silicide and at 37 K for the germanide lead to a commensurate κ_1_ = [0, 0, 0] magnetic structure having FM and AFM components. At lower temperatures, an additional AFM coupling, appearing below *T*_N2_ ∼ 24 K for the silicide and ∼22 K for the germanide and following an incommensurate magnetic propagation vector κ_2_ = [κ_*x*_, 0, 0], coexists with the first magnetic structure.

## Supporting Information Available

Single-crystal data, SEM photographs, Rietveld refinements data from powder XRD, isothermal magnetization plots of Ho_2_Ni_0.8_Si_1.2_ and Ho_2_Ni_0.8_Ge_1.2_ for data measured at 2 K, plots of the integrated neutron diffraction intensities of the (1, 0, 0) magnetic peak of both compounds, and temperature dependence of the strongest low-angle neutron diffraction peak of Ho_2_Ni_0.8_Ge_1.2_ (PDF)

## Accession Codes

CCDC 2097063 contains the supplementary crystallographic data for this paper. These data can be obtained free of charge via www.ccdc.cam.ac.uk/data_request/cif, or by emailing data_request@ccdc.cam.ac.uk, or by contacting The Cambridge Crystallographic Data Centre, 12 Union Road, Cambridge CB2 1EZ, UK; fax: +44 1223 336033.
