# Supplementary material for: Crystal and Magnetic Structures of the Ternary Ho2Ni0.8Si1.2 and Ho2Ni0.8Ge1.2 Compounds: An Example of Intermetallics Crystallizing with the Zr2Ni1–xP Prototype
Source: Inorg Chem. 2021 Oct 15;60(21):16397–408. doi: 10.1021/acs.inorgchem.1c02211 (PMC8564752; doi:10.1021/acs.inorgchem.1c02211)
Supplement: Supplementary file 1 — ic1c02211_si_001.pdf [file ic1c02211_si_001.pdf]

Crystal and magnetic structures of the ternary $Ho_2Ni_{0.8}Si_{1.2}$ and $Ho_2Ni_{0.8}Ge_{1.2}$ compounds: an example of intermetallics crystallizing with the $Zr_2Ni_{1-x}P$ prototype

Alessia Provino [a,b,#], Clemens Ritter [c,*], Volodymyr Smetana [d], Anja-Verena Mudring [d], Marcella Pani [a,e], Vitalij K. Pecharsky [b,f], Pietro Manfrinetti [a,b,e,*]

[a] *Department of Chemistry, University of Genova, 16146 Genova, Italy*

[b] *The Ames Laboratory, USDOE, Iowa State University, Ames, IA 50011-3020, USA*

[c] *Institut Laue-Langevin, 38042 Grenoble, France*

[d] *Department of Materials and Environmental Chemistry, Stockholm University, 10691 Stockholm,* Sweden

[e] *Institute SPIN-CNR, 16152 Genova, Italy*

[f] *Department of Materials Sciences and Engineering, Iowa State University, Ames, IA 50011-2300, USA*

(*) Corresponding author: ritter@ill.fr ; chimfis@chimica.unige.it

(#) Current address: Department of Chemistry and Chemical Biology, Rutgers University. Piscataway, NJ 08854, New Jersey, United States.

Supplementary Information

Single crystal data, with refinement details, and anisotropic displacement parameters for $\mathbf{Ho_2Ni_{0.769(5)}Si_{1.231(5)}}$ at T = 293(2) K are reported in **Table S1** and **Table S2**, respectively.

To study if any solid solubility was present for the phase $Ho_2Ni_{0.8}T_{1.2}$, and the eventual Ni/T solubility limits, six samples (nominally $Ho_{50}Ni_{18.3}T_{31.7}$, $Ho_{50}Ni_{20}T_{30}$ and $Ho_{50}Ni_{25}T_{25}$) were prepared for T = Si and Ge and investigated by powder XRD and SEM microscopy with EDX microprobe.

SEM photos, representative of the microstructure of the samples with nominal composition $Ho_{50}Ni_{18.3}Si_{31.7}$, $Ho_{50}Ni_{20}Si_{30}$ and $Ho_{50}Ni_{25}Si_{25}$, are shown in **Figures S1-a**, **S1-b** and **S1-c**, respectively. In $Ho_{50}Ni_{18.3}Si_{31.7}$ sample the matrix is a phase with composition $Ho_{50(1)}Ni_{18(1)}Si_{31(1)}$, while the darker grains are pertaining to Ho_3NiSi_2 [$Ho_5Ni_{0.20(4)}Si_{2.80(4)}$ (Mn_5Si_3-type) phase also detected on XRD specimen is not seen in SEM specimen]. In nominal $Ho_{50}Ni_{20}Si_{30}$ sample, the only over the three to be almost single phase material, the main phase (light matrix) is the phase with composition $Ho_{50(1)}Ni_{20(1)}Si_{30(1)}$ while the few grey grains are from equiatomic HoNiSi phase (while just a trace amount of Ho_2O_3 is detected by XRD). In the $Ho_{50}Ni_{25}Si_{25}$ sample the main

phase, as matrix, shows up with a composition $Ho_{50(1)}Ni_{22(1)}Si_{28(1)}$, while the larger delimited grains are from $HoNi_{1-x}Si_x$ (CrB-type from XRD).

The Rietveld structural refinement performed on the X-ray powder pattern of all the six samples ($Ho_{50}Ni_{18.3}T_{31.7}$, $Ho_{50}Ni_{20}T_{30}$ and $Ho_{50}Ni_{25}T_{25}$, for T = Si and Ge) corroborates the results obtained from SEM-EDX. The crystallographic data obtained from the Rietveld refinements are collected in **Tables S3** to **S5** for $Ho_{50}Ni_{18.3}Si_{31.7}$, $Ho_{50}Ni_{20}Si_{30}$ and $Ho_{50}Ni_{25}Si_{25}$ and in **Tables S6** to **S8** for $Ho_{50}Ni_{18.3}Ge_{31.7}$, $Ho_{50}Ni_{20}Ge_{30}$ and $Ho_{50}Ni_{25}Ge_{25}$, respectively.

Figure S2 highlights a comparison of the isothermal magnetization of both $\mathbf{Ho_2Ni_{0.8}Si_{1.2}}$ and $\mathbf{Ho_2Ni_{0.8}Ge_{1.2}}$ for data measured at 2 K in the range ± 70 kOe. The inset shows an enlarged view of the data between ± 4 kOe.

Figures S3-a and **S3-b** show the plots of the integrated intensities of the (1, 0, 0) magnetic peak, which represents the strongest of all magnetic peaks of the $\kappa 1$ = [0, 0, 0] phase (the first magnetic phase to appear on cooling), as a function of temperature. Both plots nicely show the T_C value of the two compounds: about 37 K for the germanide (**Fig. S3-a**) and about 38 K for the silicide (**Fig. S3-b**).

Figure S4 shows the peak intensity (**S4-a**), full width at half maximum (**S4-b**) and peak position (**S4-c**) of the very strong low-angle peak of $\mathbf{Ho_2Ni_{0.8}Ge_{1.2}}$. Small anomalies at around 13-14 K become evident.

Finally, **Table S9** gives the basis vectors (**BV**) of the allowed irreducible representations (**IR**) in $\mathbf{Ho_2Ni_{0.8}T_{1.2}}$ compounds for propagation vector κ_1 = [0,0,0], along with the Wyckoff position 4*c* of space group *Pnma*.

Table S1. Single crystal data and refinement details for $\mathbf{Ho_2Ni_{0.769(5)}Si_{1.231(5)}}$ [T = 293(2) K].

**Chemical formula**	$\mathbf{Ho_2Ni_{0.769(5)}Si_{1.231(5)}}$
Formula Weight [g/mol]	409.569
Wavelength, λ	0.71073 Å, Mo Kα
Structural prototype	$Zr_2Ni_{1-x}P$ ($Zr_2Ni_{0.48}P$)
Pearson symbol	*oP*32
Crystal system	Orthorhombic
Space group	*Pnma* (No. 62)
Lattice parameters	a = 14.883(1) Å<br>b = 4.0965(3) Å<br>c = 11.0515(8) Å
Unit cell volume [Å^3]	673.79(9)
Formula units per cell, Z	8
Calculated density, ρ [g/cm^3]	8.075
Absorption coefficient, μ [mm^{-1}]	50.9
F(000)	1382
Crystal description	Thin regular platelet
Approximate crystal size [mm^3]	0.02 × 0.08 × 0.09
Theta range for data collection [deg]	2.3° ≤ θ ≤ 32.5°
h, k, l ranges	$-22 \leq h \leq 22$, $-6 \leq k \leq 6$, $-15 \leq l \leq 16$
Reflections collected	15342
Independent reflections	1366
Absorption correction	Empirical
Refinement method	Full-matrix least-squares on F^2
Data/restraints/parameters	1366/0/52
Goodness of fit on F^2	1.10
Final R indices [$I > 2\sigma(I)$]	$R1 = 0.0275$, $wR^2 = 0.0430$
R indices (all data)	$R1 = 0.0395$, $wR^2 = 0.0444$
R_{int}	0.078
Largest diff. peak and hole [e$^-$/Å^3]	+2.4, –2.2

Table S2. Anisotropic displacement parameters (U_{ij}) of $\mathbf{Ho_2Ni_{0.769(5)}Si_{1.231(5)}}$; $U_{23} = U_{12} = 0$.

Atom	Wyckoff site	U_{11} [Å^2]	U_{22} [Å^2]	U_{33} [Å^2]	U_{13} [Å^2]
Ho1	4*c*	0.0070(2)	0.0065(2)	0.0070(2)	0.0007(2)
Ho2	4*c*	0.0074(2)	0.0070(2)	0.0060(2)	0.0004(2)
Ho3	4*c*	0.0063(2)	0.0064(2)	0.0080(2)	–0.0014(15)
Ho4	4*c*	0.0060(2)	0.0055(2)	0.0059(2)	0.0001(1)
Ni1	4*c*	0.0076(5)	0.0051(6)	0.0096(5)	–0.0019(4)
Ni2/Si	4*c*	0.0082(8)	0.0058(9)	0.0075(8)	0.0012(6)
Si1/Ni	4*c*	0.0088(12)	0.0063(14)	0.0074(13)	–0.0004(9)
Si2	4*c*	0.0059 (11)	0.0057(12)	0.0100(12)	–0.0034(9)

Table S3. Data of the Rietveld refinement for the sample with nominal composition $Ho_{50}Ni_{18.3}Si_{31.7}$ (R_{wp} = 8.17 %, χ^2 = 2.30).

Atom	Wyckoff site	Atomic coordinates			B_{iso} [Å^2]	Occupancy
		x	*y*	*z*		
Ho1	4*c*	0.0336(1)	1/4	0.7860(2)	1.65(6)	1
Ho2	4*c*	0.1478(1)	1/4	0.0922(2)	1.44(6)	1
Ho3	4*c*	0.2703(1)	1/4	0.3568(2)	1.54(6)	1
Ho4	4*c*	0.3942(1)	1/4	0.0295(2)	1.46(5)	1
Ni1	4*c*	0.3461(3)	1/4	0.7798(4)	1.1(1)	1
Ni2/Si	4*c*	0.45599(7)	1/4	0.47319(9)	0.4(1)	0.501/0.499(2)
Si1	4*c*	0.0712(5)	1/4	0.3410(8)	0.7(2)	1
Si2	4*c*	0.2187(5)	1/4	0.6587 (7)	0.6(2)	1

Final refined stoichiometry of the main phase: $\mathbf{Ho_2Ni_{0.750(1)}Si_{1.250(1)}}$ = $Ho_{50}Ni_{18.75(2)}Si_{31.25(2)}$

Phase amount: 64.4(3) vol.%

a = 14.9136(1) Å, b = 4.0986(1) Å, c = 11.0688(1) Å, V_{obs} = 676.571(3) Å^3

R_B = 1.96 %, R_F = 1.75 %

Impurity phase (1): $\mathbf{Ho_3NiSi_2}$ (Gd_3NiSi_2-type, *oP*24, *Pnma*, No. 62)

Phase amount: 34.5(2) vol. %

a = 11.1955(1) Å, b = 4.1074(1) Å, c = 11.1150(1) Å, V_{obs} = 511.127(3) Å^3

R_B = 1.78 %, R_F = 1.64 %

Impurity phase (2) refined stoichiometry: $\mathbf{Ho_5Ni_{0.20(4)}Si_{2.80(4)}}$ (Mn_5Si_3-type, *hP*16, $P6_3/mcm$, No. 193)

Phase amount: 1.12(5) vol. %

a = 8.3468(2) Å, c = 6.2824(2) Å, V_{obs} = 379.05(2) Å^3

R_B = 3.97 %, R_F = 3.50 %

Table S4. Data of the Rietveld refinement for the sample with nominal composition $Ho_{50}Ni_{20}Si_{30}$ (R_{wp} = 7.98 %, χ^2 = 2.11).

**Atom**	**Wyckoff site**	**Atomic coordinates**			**B_{iso} [Å^2]**	**Occupancy**
		x	***y***	***z***		
Ho1	4*c*	0.03361(8)	1/4	0.7876(1)	1.70(3)	1
Ho2	4*c*	0.14694(9)	1/4	0.0937(1)	1.68(3)	1
Ho3	4*c*	0.27091(8)	1/4	0.3570(1)	1.70(4)	1
Ho4	4*c*	0.39321(9)	1/4	0.0300(1)	1.65(3)	1
Ni1	4*c*	0.3485(2)	1/4	0.7825(2)	0.94(7)	1
Ni2/Si	4*c*	0.45688(5)	1/4	0.47239(7)	1.15(9)	0.548/0.452(1)
Si1/Ni	4*c*	0.07194(6)	1/4	0.34273(9)	1.3(1)	0.855/0.145(1)
Si2	4*c*	0.2183(3)	1/4	0.6583(5)	1.1(1)	1

Final refined stoichiometry of the main phase: $\mathbf{Ho_2Ni_{0.846(1)}Si_{1.154(1)}}$ = $Ho_{50}Ni_{21.15(2)}Si_{28.85(2)}$

Phase amount: 99.2(2) vol.%

a = 14.8988(1) Å, b = 4.0996(1) Å, c = 11.0606(1) Å, V_{obs} = 675.579(2) Å^3

R_B = 1.98 %, R_F = 2.31 %

Impurity phase: $\mathbf{Ho_2O_3}$ (Mn_2O_3-type, *cI*80, *Ia*–3, No. 206)

Phase amount: 0.79(7) vol. %

a = 10.6117(1) Å, V_{obs} = 1194.95(3) Å^3

R_B = 7.61 %, R_F = 5.04

Table S5. Data of the Rietveld refinement for the sample with nominal composition $Ho_{50}Ni_{25}Si_{25}$ (R_{wp} = 7.00 %, χ^2 = 1.84).

Atom	Wyckoff site	Atomic coordinates			B_{iso} [Å^2]	Occupancy
		x	*y*	*z*		
Ho1	4*c*	0.03386(9)	1/4	0.7866(1)	1.22(4)	1
Ho2	4*c*	0.1472(1)	1/4	0.0943(1)	0.98(4)	1
Ho3	4*c*	0.2708(1)	1/4	0.3554(1)	0.89(4)	1
Ho4	4*c*	0.3931(1)	1/4	0.0293(1)	0.91(4)	1
Ni1	4*c*	0.3480(2)	1/4	0.7810(3)	0.60(8)	1
Ni2/Si	4*c*	0.4573(2)	1/4	0.4681(3)	0.3(1)	0.663/0.337(2)
Si1/Ni	4*c*	0.0698(4)	1/4	0.3437(5)	0.6(2)	0.968/0.032(2)
Si2/Ni	4*c*	0.2119(3)	1/4	0.6537(5)	0.7(1)	0.852/0.148(2)

Final refined stoichiometry of the main phase: $\mathbf{Ho_2Ni_{0.922(3)}Si_{1.078(3)}}$ = $Ho_{50}Ni_{23.05(8)}Si_{26.95(8)}$

Phase amount: 82.2(2) vol.%

a = 14.8798(1) Å, b = 4.0995(1) Å, c = 11.0550(1) Å, V_{obs} = 674.349(3) Å^3

R_B = 1.20 %, R_F = 1.17 %

Impurity phase refined stoichiometry: $\mathbf{HoNi_{0.855(2)}Si_{0.145(2)}}$ (CrB-type, *oS*8, *Cmcm*, No. 63)

Phase amount: 17.84(8) vol. %

a = 3.7633(1) Å, b = 10.2841(2) Å, c = 4.1379(1) Å, V_{obs} = 160.148(4) Å^3

R_B = 0.87 %, R_F = 0.66 %

Table S6. Data of the Rietveld refinement performed on the sample with nominal composition $Ho_{50}Ni_{18.3}Ge_{31.7}$ (R_{wp} = 7.35 %, χ^2 = 2.11).

Atom	Wyckoff site	Atomic coordinates			B_{iso} [Å^2]	Occupancy
		x	*y*	*z*		
Ho1	4*c*	0.0348(1)	1/4	0.7889(2)	1.82(6)	1
Ho2	4*c*	0.1463(1)	1/4	0.0922(2)	1.86(6)	1
Ho3	4*c*	0.2680(1)	1/4	0.3542(2)	1.59(5)	1
Ho4	4*c*	0.3930(1)	1/4	0.0270(2)	1.44(5)	1
Ni1	4*c*	0.3480(3)	1/4	0.7788(4)	1.1(1)	1
Ni2/Ge	4*c*	0.45211(4)	1/4	0.46911(6)	0.63(2)	0.598/0.402(2)
Ge1	4*c*	0.0676(2)	1/4	0.3415(3)	0.68(8)	1
Ge2	4*c*	0.2134(2)	1/4	0.6545(3)	0.63(8)	1

Final refined stoichiomtery of the main phase: $\mathbf{Ho_2Ni_{0.799(1)}Ge_{1.201(1)}}$ = $Ho_{50}Ni_{19.98(2)}Ge_{30.02(2)}$

Phase amount: 59.5(2) vol.%

a = 15.0138(1) Å, b = 4.1484(1) Å, c = 11.0753(1) Å, V_{obs} = 689.804(9) Å^3

R_B = 2.05 %, R_F = 1.85 %

Impurity phase: $\mathbf{Ho_3NiGe_2}$ (Gd_3NiSi_2-type, *oP*24, *Pnma*, No. 62)

Phase amount: 39.7(1) vol. %

a = 11.2922(1) Å, b = 4.1542(1) Å, c = 11.1410(1) Å, V_{obs} = 522.62(2) Å^3

R_B = 1.86 %, R_F = 2.04 %

Impurity phase: **HoNiGe** (TiNiSi-type, *oP*12, *Pnma*, No. 62)

Phase amount: 0.78(3) vol. %

a = 6.849(2) Å, b = 4.1836(7) Å, c = 7.312(1) Å, V_{obs} = 209.5(1) Å^3

R_B = 6.09 %, R_F = 1.92 %

Table S7. Data of the Rietveld refinement performed on the sample with nominal composition $Ho_{50}Ni_{20}Ge_{30}$ (R_{wp} = 7.40 %, χ^2 = 2.04).

Atom	Wyckoff site	Atomic coordinates			B_{iso} [Å^2]	Occupancy
		x	*y*	*z*		
Ho1	4*c*	0.03527(7)	1/4	0.7898(1)	1.86(3)	1
Ho2	4*c*	0.14610(8)	1/4	0.0941(1)	0.83(3)	1
Ho3	4*c*	0.26854(8)	1/4	0.3548(1)	1.80(3)	1
Ho4	4*c*	0.39336(8)	1/4	0.0270(1)	1.64(3)	1
Ni1	4*c*	0.3472(2)	1/4	0.7780(2)	1.50(7)	1
Ni2/Ge	4*c*	0.45261(3)	1/4	0.46871(4)	1.10(6)	0.609/0.391(2)
Ge1	4*c*	0.0669(1)	1/4	0.3434(2)	0.97(5)	1
Ge2	4*c*	0.2134(1)	1/4	0.6535(2)	0.90(5)	1

Final refined stoichiomtery: $\mathbf{Ho_2Ni_{0.804(1)}Ge_{1.196(1)}}$ = $Ho_{50}Ni_{20.109(1)}Ge_{29.891(1)}$

a = 15.0054(1) Å, b = 4.1483(1) Å, c = 11.0719(1) Å, V_{obs} = 689.191(5) Å^3

R_B = 1.70 %, R_F = 2.55 %

No impurity phases.

Table S8. Data of the Rietveld refinement performed on the sample with nominal composition $Ho_{50}Ni_{25}Ge_{25}$ (R_{wp} = 6.68 %, χ^2 = 1.71).

Atom	Wyckoff site	Atomic coordinates			B_{iso} [Å^2]	Occupancy
		x	*y*	*z*		
Ho1	4*c*	0.03447(2)	1/4	0.78984(2)	1.807(2)	1
Ho2	4*c*	0.14510(2)	1/4	0.09315(2)	1.922(2)	1
Ho3	4*c*	0.26821(7)	1/4	0.35343(2)	1.997(2)	1
Ho4	4*c*	0.39311(2)	1/4	0.02666(2)	1.647(2)	1
Ni1	4*c*	0.34764(4)	1/4	0.77916(5)	1.325(4)	1
Ni2/Ge	4*c*	0.45411(3)	1/4	0.46972(4)	1.12(1)	0.620/0.380(1)
Ge1	4*c*	0.06792(3)	1/4	0.34220(4)	0.587(3)	1
Ge2	4*c*	0.21439(3)	1/4	0.65319(4)	0.991(3)	1

Final refined stoichiomtery of the main phase: $\mathbf{Ho_2Ni_{0.810(1)}Ge_{1.190(1)}}$ = $Ho_{50}Ni_{19.25(2)}Ge_{29.75(2)}$

Phase amount: 85.4(2) vol.%

a = 14.9824(1) Å, b = 4.1468(1) Å, c = 11.0696(1) Å, V_{obs} = 687.748(7) Å^3

R_B = 1.04 %, R_F = 1.15 %

Minority phase final stoichiometry: $\mathbf{HoNi_{0.925}Ge_{0.075}}$ (FeB-type, *oP*8, *Pnma*, No. 62) (composition as from EDX)

Phase amount: 14.6(1) vol. %

a = 7.0288(1) Å, b = 4.1579(1) Å, c = 5.4476(1) Å, V_{obs} = 159.206(9) Å^3

R_B = 0.78 %, R_F = 0.77 %

Table S9.

Basis vectors (**BV**) of the allowed irreducible representations (**IR**) in $\mathbf{Ho_2Ni_{0.8}T_{1.2}}$, for κ_1 = [0,0,0] and the Wyckoff position 4*c* of space group *Pnma*.

	**IR1**	**IR2**		**IR3**		**IR4**	**IR5**	**IR6**		**IR7**		**IR8**
4*c*		**BV1**	**BV2**	**BV1**	**BV2**			**BV1**	**BV2**	**BV1**	**BV2**	
x,y,z	0 1 0	1 0 0	0 0 1	1 0 0	0 0 1	0 1 0	0 1 0	1 0 0	0 0 1	1 0 0	0 0 1	0 1 0
-*x*+½, -*y*, *z*+½	0 -1 0	-1 0 0	0 0 1	-1 0 0	0 0 1	0 -1 0	0 1 0	1 0 0	0 0 -1	1 0 0	0 0 -1	0 1 0
-*x*, *y*+½, -*z*	0 1 0	-1 0 0	0 0 -1	1 0 0	0 0 1	0 -1 0	0 1 0	-1 0 0	0 0 -1	1 0 0	0 0 1	0 -1 0
x+½, -*y*+½, -*z*+½	0 -1 0	1 0 0	0 0 -1	-1 0 0	0 0 1	0 1 0	0 1 0	-1 0 0	0 0 1	1 0 0	0 0 -1	0 -1 0

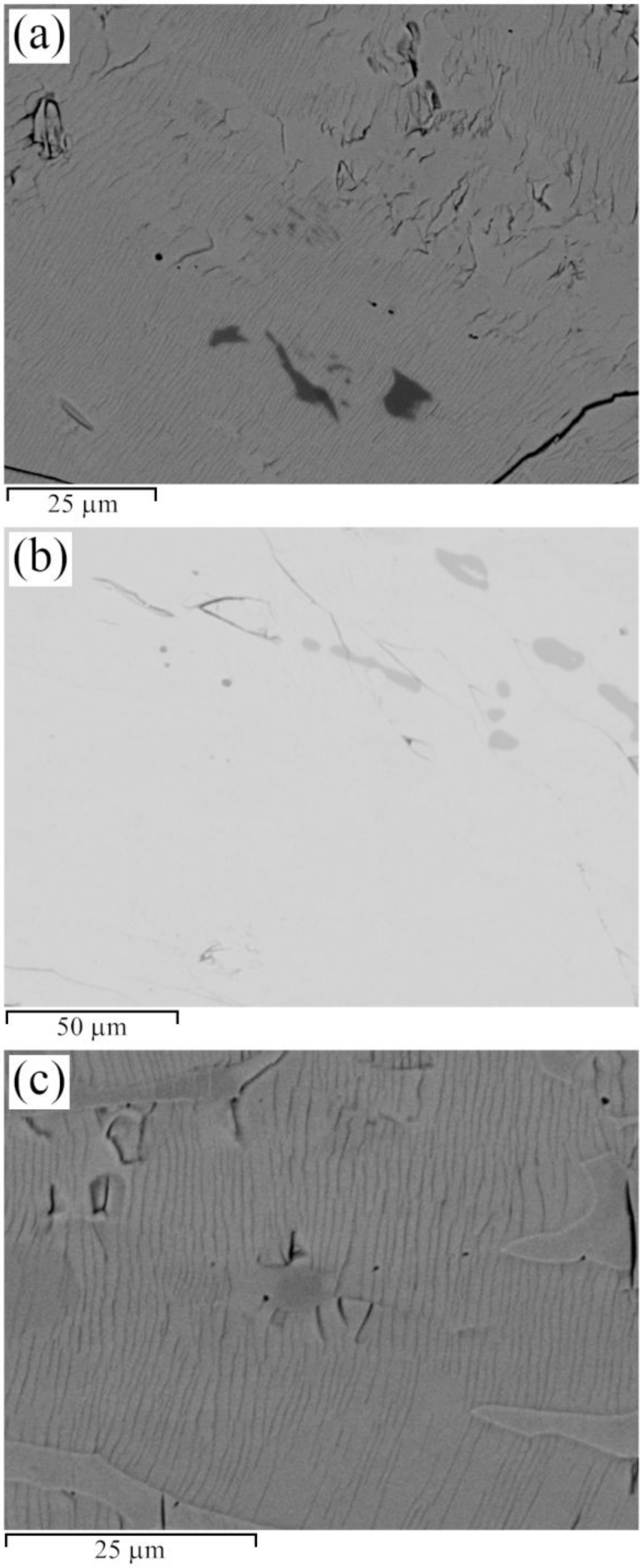
(a)
25 μm
(b)
50 μm
(c)
25 μm

Figure S1. SEM microphotographs (BSE mode) showing the microstructure of the annealed samples with nominal composition $Ho_{50}Ni_{18.3}Si_{31.7}$ (**S1-a**), $Ho_{50}Ni_{20}Si_{30}$ (**S1-b**) and $Ho_{50}Ni_{25}Si_{25}$ (**S1-c**). In $Ho_{50}Ni_{18.3}Si_{31.7}$ sample the matrix is a phase with composition $Ho_{50(1)}Ni_{18(1)}Si_{31(1)}$, while the darker grains are Ho_3NiSi_2. $Ho_5Ni_{0.20(4)}Si_{2.80(4)}$ (Mn_5Si_3-type) phase detected with XRD is not seen in this SEM specimen. In the nominal $Ho_{50}Ni_{20}Si_{30}$ sample, the only over the three to be almost single phase material, the main phase (light matrix) is the phase with composition $Ho_{50(1)}Ni_{20(1)}Si_{30(1)}$ while a few grey grains are from equiatomic HoNiSi phase. A trace amount of Ho_2O_3 detected by XRD is not seen. In the $Ho_{50}Ni_{25}Si_{25}$ sample the main phase has composition $Ho_{50(1)}Ni_{22(1)}Si_{28(1),}$ while the larger delimited grains are from $HoNi_{1-x}Si_x$, which is CrB-type from XRD.

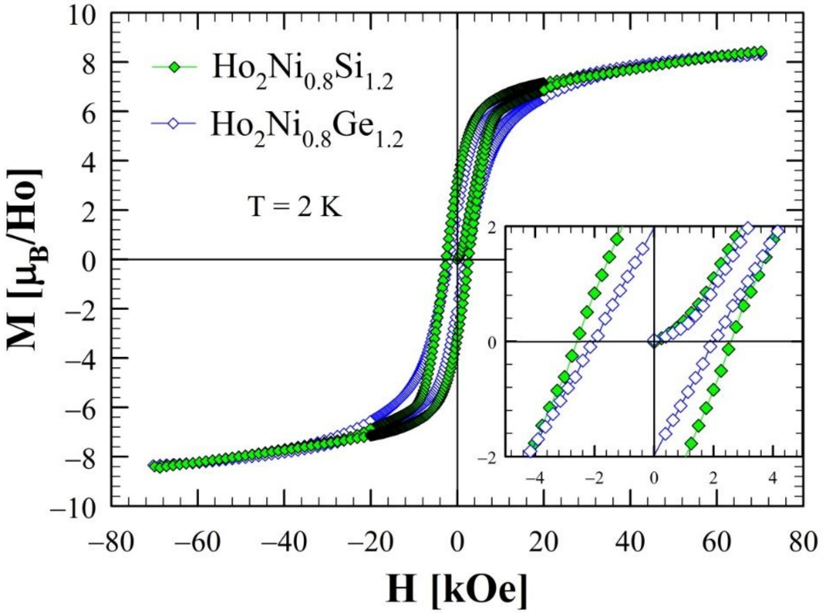

Figure S2. Comparison of isothermal magnetization of both $\mathbf{Ho_2Ni_{0.8}Si_{1.2}}$ and $\mathbf{Ho_2Ni_{0.8}Ge_{1.2}}$ for data measured at 2 K in the range ± 70 kOe. Inset shows an enlarged view of the data between ± 4 kOe.

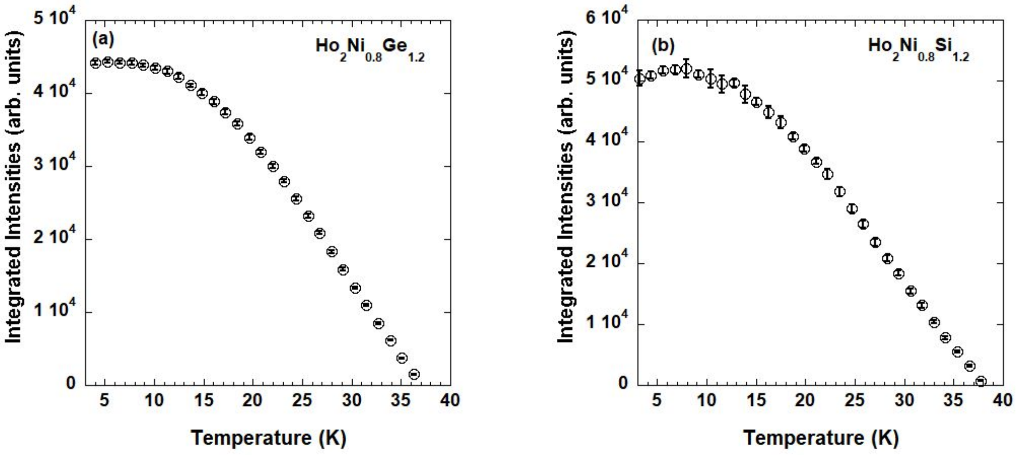

Figure S3. Integrated intensity as a function of temperature of the (1, 0, 0) magnetic peak, the strongest of all magnetic peaks of the $\kappa 1$ = [0, 0, 0] phase (the first magnetic phase appearing on cooling), for $Ho_2Ni_{0.8}Ge_{1.2}$ (**Fig. S3-a**) and $Ho_2Ni_{0.8}Si_{1.2}$ (**Fig. S3-b**).

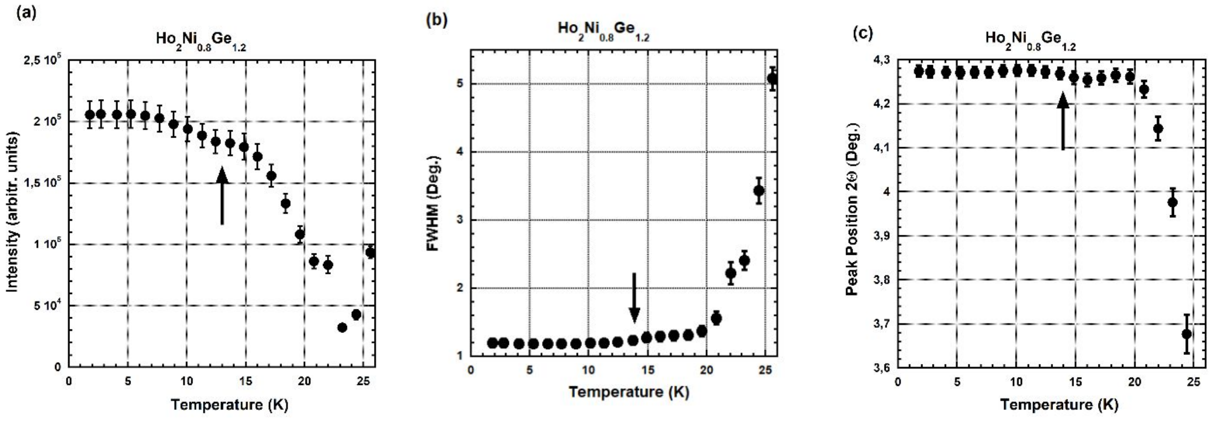

Figure S4. Peak intensity (**S4-a**), full width at half maximum (**S4-b**) and peak position (**S4-c**) of the very strong low-angle peak of $\mathbf{Ho_2Ni_{0.8}Ge_{1.2}}$. Small anomalies can be seen at around 13-14 K.
